# Targeting oxidative pentose phosphate pathway prevents recurrence in mutant *Kras* colorectal carcinomas

**DOI:** 10.1371/journal.pbio.3000425

**Published:** 2019-08-28

**Authors:** WenChao Gao, YuTing Xu, Tao Chen, ZunGuo Du, XiuJuan Liu, ZhiQian Hu, Dong Wei, ChunFang Gao, Wei Zhang, QingQuan Li

**Affiliations:** 1 Department of Pathology, School of Basic Medical Sciences, Fudan University, Shanghai, China; 2 Department of General Surgery, ChangZheng Hospital, Second Military Medical University, Shanghai, China; 3 Endoscopy Center, ZhongShan Hospital, Fudan University, Shanghai, China; 4 Department of Pathology, HuaShan Hospital, Fudan University, Shanghai, China; 5 Department of Anus and Intestine Surgery, PLA Central Hospital 150, Luoyang, China; 6 Department of Cancer Biology, Cancer Center of Wake Forest Baptist Medical Center, Winston-Salem, North Carolina, United States of America; B.C. Cancer Agency, CANADA

## Abstract

Recurrent tumors originate from cancer stem cells (CSCs) that survive conventional treatments. CSCs consist of heterogeneous subpopulations that display distinct sensitivity to anticancer drugs. Such a heterogeneity presents a significant challenge in preventing tumor recurrence. In the current study, we observed that quiescent CUB-domain–containing protein 1 (CDCP1)+ CSCs are enriched after chemotherapy in mutant Kirsten rat sarcoma viral oncogene homolog (*Kras*) colorectal carcinomas (CRCs) and serve as a reservoir for recurrence. Mechanistically, glucose catabolism in CDCP1+ CSCs is routed to the oxidative pentose phosphate pathway (PPP); multiple cycling of carbon backbones in the oxidative PPP potentially maximizes NADPH reduction to counteract chemotherapy-induced reactive oxygen species (ROS) formation, thereby allowing CDCP1+ CSCs to survive chemotherapeutic attack. This is dependent on silent mating type information regulation 2 homolog 5 (Sirt5)-mediated inhibition of the glycolytic enzyme triosephosphate isomerase (TPI) through demalonylation of Lys56. Blocking demalonylation of TPI at Lys56 increases chemosensitivity of CDCP1+ CSCSs and delays recurrence of mutant *Kras* CRCs in vivo. These findings pinpoint a new therapeutic approach for combating mutant *Kras* CRCs.

## Abstract 2

This study shows that CDCP1-positive cancer stem cells represent a critical driving force behind tumor relapse in colorectal tumors that have mutant Kras and reveals a unique role for the oxidative pentose phosphate pathway in this process.

## Introduction

Advances in pharmaceutical and surgical intervention have significantly improved the outcome in patients with colorectal carcinomas (CRCs) [1]. However, recurrence eventually occurs in nearly all patients who initially respond to systemic treatments [2]. Increasing evidence suggest that cancer stem cells (CSCs) are enriched after treatment because of resistance to anticancer cytotoxic drugs and are responsible for recurrence [3]. CSCs are an intrinsically heterogeneous population that consists of phenotypically and functionally different subsets [4]. Not all CSC subpopulations play distinct roles in drug resistance. For example, only CSCs that express aldehyde dehydrogenase 1 family member A1 (ALDH1A1) in lung cancer are resistant to gefitinib [5]. In murine CRC models, chemotherapeutic agents enrich epithelial-specific antigen (ESA)+ cluster of differentiation 44 (CD44)+ CD166+ CSCs in tumor xenografts [6]. Therefore, treatment of relapsed tumors using the regimen that results in remission of the primary tumor is suboptimal.

We previously demonstrated that colon CSCs characterized by CDCP1 expression exhibit decreased sensitivity to 5-fluoropyrimidine (5-FU) when compared with other CSC subpopulations [7]. CDCP1 is a type I transmembrane glycoprotein that consists of a 29-residue amino terminal signal peptide, an extracellular domain containing three complement C1r/C1s, Uegf, Bmp1 (CUB) domains, a transmembrane domain, and a cytoplasmic domain containing five tyrosine residues. Up-regulation of CDCP1 in a variety of solid cancers has been associated with disease progression and poor patient survival [8]. Recent literature shows that hypoxia-inducible factor (HIF)-2α is essential for induction of CDCP1 expression under hypoxic conditions, a common microenvironment for cancer cells that contributes to chemoresistance [9]. CDCP1 facilitates proto-oncogene tyrosine-protein kinase SRC (SRC)-human epidermal growth factor receptor 2 (HER2) crosstalk and confers resistance to trastuzumab treatment [10]. Based on the aforementioned facts, we speculated that colon CDCP1+ CSCs survive exposure to anticancer cytotoxic agents. http://www.tandfonline.com/doi/full/10.4161/cc.29460 - R4#R4Of note, CRC patients harboring *Kras* mutations are more likely to present with or to develop lung metastasis [11]. After curative treatment, the cumulative recurrence of lung metastases was significantly higher in CRC patients with mutant *Kras* (mu*Kras*) compared with those with wild-type Kras (wt*Kras*) [12]. Since CDCP1+ CSCs have an extremely high propensity to metastasize to the lungs [7], selective enrichment of CDCP1+ cells following chemotherapy in mu*Kras* CRCs was observed in the current study, suggesting the role of CDCP1+ CSCs in eventual recurrence for that subtype of CRCs.

To define fundamental aspects of CDCP1+ CSC biology, which are conserved irrespective of interpatient or intrapatient heterogeneity, we investigated energy metabolism, a basic requirement of any cell type. Cancer cells appear to commonly acquire aberrancies in energy metabolism [13]. For example, cancer cells in the presence of oxygen tend to employ glycolysis instead of aerobic mitochondrial respiration to generate energy [14]. However, while such tumor-related metabolic adaptations characterize bulk tumor cells, it is unclear whether colon CDCP1+ CSCs exhibit a similar metabolic phenotype. Gaining further insights into the metabolic properties of CDCP1+ CSCs may reveal physiological dependencies that can be targeted for clinical therapy.

## Results

### CDCP1+ CSCs in mu*Kras* CRCs are selectively enriched following chemotherapy

**Fig 1:**
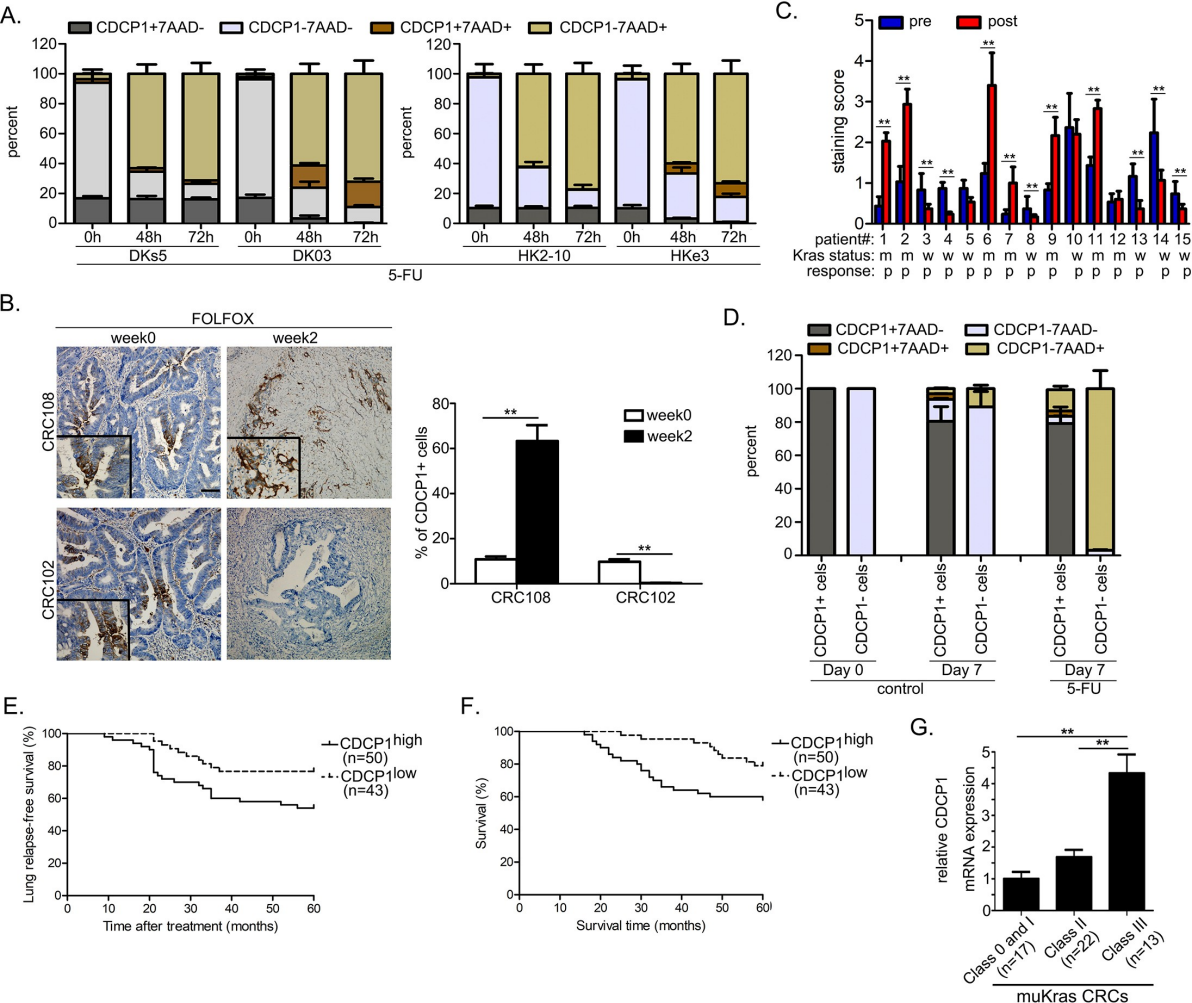
CDCP1+ CSCs in mu*Kras* CRCs are enriched following chemotherapy. (A) DKs5, DKO3, HK2-10, and HKe3 cells were persistently treated with 5-FU (5 μM) and analyzed for CDCP1 expression by flow cytometry at the indicated time points. Dead cells were detected by 7AAD staining. The experiments were independently repeated three times in triplicate. (B) BALB/c-nu mice bearing CRC108 or CRC102 epidermal xenografts received FOLFOX treatment twice weekly for 2 weeks. Representative images (left panels) and quantification (right panel) of CDCP1+ cell frequency in untreated tumors (week 0; *n* = 3) and tumor remnants after FOLFOX (week 2; *n* = 3) were evaluated by IHC. (C) Comparison of immunohistochemical CDCP1 staining from 15 CRC patients before and after neoadjuvant therapy. All patients achieved a partial response (P). (D) CRC108 cells were FACS-sorted according to their CDCP1 expression, and the isolated subfractions were cultured as adherent monolayers overnight. After subsequent 5-FU treatment (5 μM) for 7 days, the frequencies of CDCP1+ and CDCP1– cells were again quantified by flow cytometry. Dead cells were detected by 7AAD staining. The experiments were independently repeated three times in triplicate. (E–F) Kaplan–Meier graphs showing the fraction of patients with lung-recurrence–free survival (E) or overall survival (F) for the patients with mu*Kras* CRCs, dichotomized by CDCP1 expression status of primary tumors. *P*-values are determined by the log-rank test. (G) CDCP1 expression in primary mu*Kras* CRCs prior to treatment versus RCB. Sample size is indicated in parentheses. Values shown are mean ± SD. A two-tailed unpaired *t* test was used to compare experimental groups. **p < 0.05. Bars: 50 μm. Underlying data are available in S1 Data. CDCP1, CUB-domain–containing protein 1; CRC, colorectal carcinoma; CSC, cancer stem cell; FACS, fluorescence-activated cell sorting; FOLFOX, folinic acid + fluorouracil + oxaliplatin; IHC, immunohistochemistry; *Kras*, Kirsten rat sarcoma viral oncogene homolog; mu*Kras*, mutant *Kras*; RCB, residual cancer burden; 5-FU, 5-fluoropyrimidine; 7AAD, 7-aminoactinomycin.

In this study, we used a panel of isogenic CRC cell lines, DLD1-derived DKs5 (*Kras*^*G13D*^) and DKO3 (*Kras*^*WT*^) cells and HCT116-derived HK2-10 (*Kras*^*G13D*^) and HKe3 (*Kras*^*WT*^) cells, in which wt*Kras* or mu*Kras* alleles have been disrupted by targeted homologous recombination [15]. When cell lines with mu*Kras* were treated with 5-FU for 48 and 72 h, a gradual separation of the total population emerged into live 7-aminoactinomycin–(7AAD–) and dead 7AAD+ subpopulations. With increasing incubation time, 7AAD–cells were incrementally enriched for CDCP1+ cells, whereas 7AAD+ cells were relatively negative for CDCP1 (Fig 1A). Oxaliplatin also failed to eradicate all mu*Kras* CRC cells and enriched for CDCP1+ cells, however, to a lesser extent than seen for 5-FU (S1A Fig). By contrast, cell lines harboring wt*Kras* did not show such an enrichment pattern upon treatment of chemotherapeutic agents (5-FU and oxaliplatin) or anti-epidermal growth factor receptor (EGFR) drugs (cetuximab) (Fig 1A; S1A and S1B Fig), indicating other potential operating mechanisms of drug resistance.

The in vitro observations were confirmed in mice bearing patient-derived epidermal xenografts (CRC102 with *Kras*^*WT*^; CRC108 with *Kras*^*G12D*^) administrated with folinic acid + fluorouracil +oxaliplatin (FOLFOX) regimen (5-FU, leucovorin, and oxaliplatin). The treatment resulted in rapid tumor shrinkage within 2 weeks. Tumors relapsed within 4 weeks of treatment cessation in all animal subjects (S1C Fig). Notably, CDCP1+ cells were selectively enriched in epithelial remnants of CRC108 but not CRC102 tumors (Fig 1B). In addition, we examined changes in CDCP1 staining after neoadjuvant treatment in 15 CRC patients (7 cases with mu*Kras*, 8 cases with wt*Kras*), all of whom initially achieved a partial regression to drug treatment. As compared to the pretreatment values, the score of CDCP1 staining was significantly increased in 6 out of 7 residual tumors with mu*Kras* (Fig 1C). These data imply a universal chemoresistance of CDCP1+ CSCs in mu*Kras* CRCs.

Next, we determined whether the drug-induced enrichment of CDCP1+ cells results from selection of pre-existing cells or induction of CDCP1 expression. As shown in Fig 1D, about 15.7% of sorted CDCP1+ cells turned to be CDCP1-negative within 7 days after separation, while no sorted CDCP1– cells became CDCP1-positive, indicating one-way phenotypic shift between the two subsets. After treatment with 5-FU for 7 days, we saw no substantial enrichment for CDCP1+ cells in the progeny of initially CDCP1+ cells, suggesting that no additional CDCP1+ cells were induced. Most newly developed CDCP1– cells underwent apoptosis under 5-FU treatment. Meanwhile, no apparent preference for CDCP1 induction in the progeny of CDCP1– cells was observed. Together with the results from consecutive measurements of CDCP1 mRNA under 5-FU treatment that did not show transcriptional up-regulation (S1D Fig), we concluded that the major mechanism for the enrichment of CDCP1+ cells following chemotherapy is a selection of pre-existing CDCP1+ CSCs.

### High CDCP1 expression in mu*Kras* CRCs correlates with shorter time to lung recurrence and poorer response to neoadjuvant chemotherapy

We further asked the value of CDCP1 expression in predicting recurrence and therapeutic responses in mu*Kras* CRCs. In the first cohort of 93 stage II or III primary mu*Kras* CRC patients receiving curative treatments, recurrence was noted in 52 subjects within 5 years (21 in the liver, 31 in the lungs, and 2 in both sites). Analyzing the data by dividing CDCP1 expression in primary CRCs prior to chemotherapy (CDCP1^high^ versus CDCP1^low/negative^; S1E Fig) suggested an association of high CDCP1 expression with shorter time to lung recurrence (Fig 1E) and poor overall survival (Fig 1F). High CDCP1 expression was not associated with relapse in the liver (S1F Fig). After adjusting for age, gender, tumor stage, site, and standard therapy in a multivariate analysis, high CDCP1 expression was an independent risk factor for lung recurrence (S1 Table).

In a separate cohort of 52 cases with stage II or III mu*Kras* CRCs, we examined the relationship between patient responses to neoadjuvant chemotherapy and CDCP1 mRNA level using prechemotherapeutic tumor biopsies. For this purpose, residual cancer burden (RCB) was assessed at the time of surgery, which takes into account parameters such as tumor bed area, overall cellularity, and lymph node involvement. Tumors with the poorest response to neoadjuvant chemotherapy (RCB III) exhibited substantially higher CDCP1 expression prior to therapy than tumors that exhibited a greater response to chemotherapy (RCB 0, I, and II) (Fig 1G). By stark contrast, CDCP1 expression did not portend a poorer response to neoadjuvant chemotherapy in a pool of 47 stage II or III CRCs harboring wt*Kras* (S1G Fig).

### CDCP1+ CSCs drive post-therapeutic recurrence in mu*Kras* CRCs

**Fig 2:**
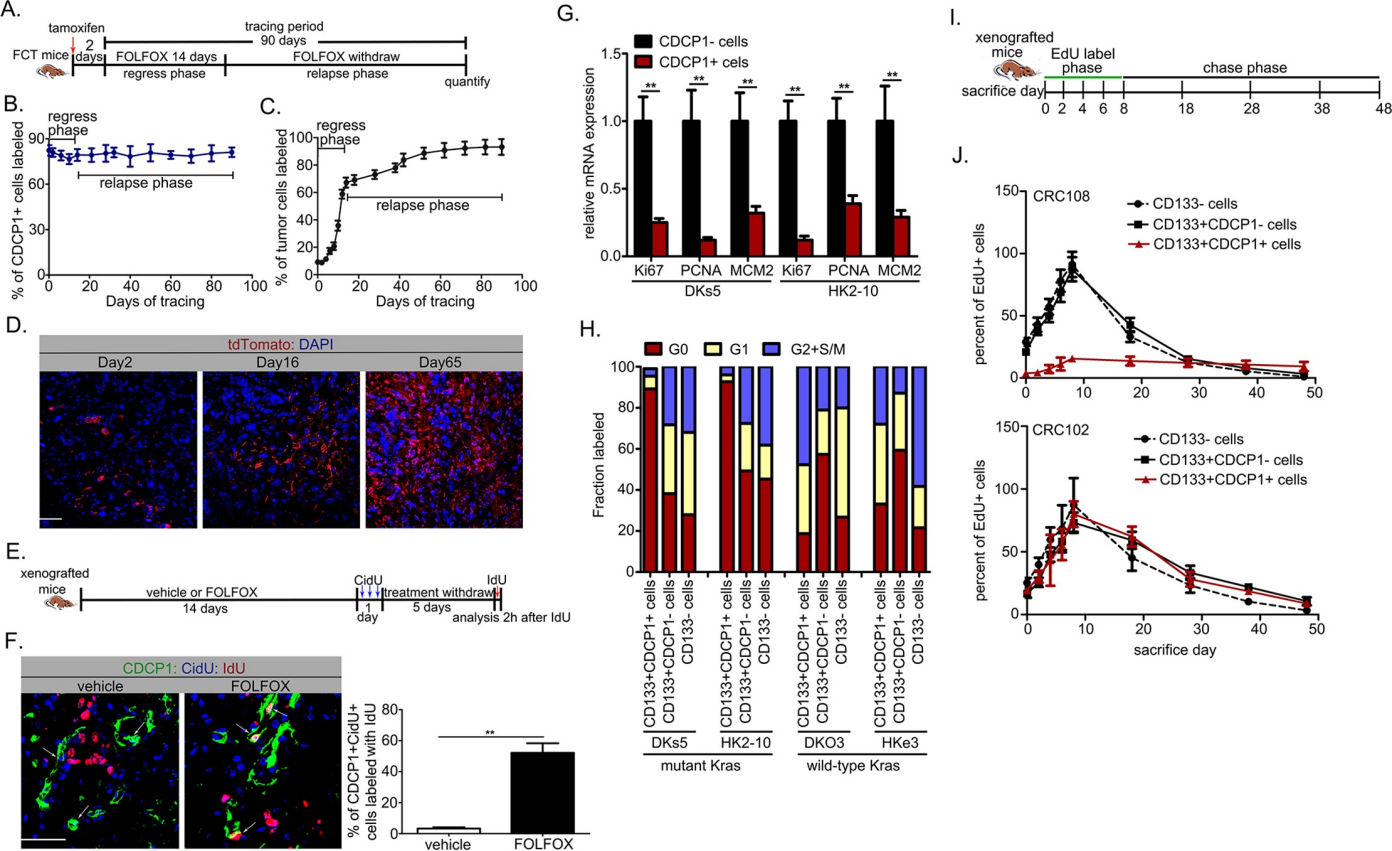
Quiescent CDCP1+ CSCs contribute to postchemotherapeutic recurrence in mu*Kras* CRCs. (A) Design for lineage tracing experiments. FCT mice (*n* = 55) received 5 mg tamoxifen 48 h prior to FOLFOX administration, after which tumor regression and relapse were monitored for 90 days. (B) The frequency of tdTomato+ cells also positive for CDCP1 post-tamoxifen injection (*n* = 3 per time point). (C) Quantification of tdTomato labeling of tumor cells following tamoxifen injection (*n* = 3 per time point). (D) Representative images of tumor labeling with tdTomato at day 2, 16, and 65 post-tamoxifen injection. (E) Experimental design. NOG mice were randomized to receive FOLFOX (*n* = 5) or vehicle treatment (*n* = 5) twice weekly for two weeks when GFP-labeled CRC101 orthotopic xenografts reached approximately 250 units under intravital imaging. Mice received 3 CidU injections (at 2 h interval) on day 1 after FOLFOX or vehicle treatment. Five days later, mice received IdU 2 h before being killed. (F) Representative images (left panels) and quantification (right panel) of a CDCP1+ CidU+ cell labeled with IdU in tumor sections from Fig 3E. (G) Representative markers for proliferation were examined at the mRNA level in CDCP1+ and CDCP1– subsets from DKs5 and HK2-10 cells. The experiments were independently repeated three times in triplicate. (H) Cell-cycle distribution of CD133+ CDCP1+, CD133+ CDCP1–, or CD133– fractions from mu*Kras* and wt*Kras* CRC cells was determined by combined staining with Hoechst33342 and pyronin Y. The experiments were independently repeated three times in triplicate. (I) Design for EdU label-chase experiments. NOG mice carrying CRC102 or CRC108 orthotopic xenografts (*n* = 30 per group) were exposed to EdU (0.82 mg/ml in drinking water) for 8 days, followed by a 40-day chase. (J) The frequency of EdU+ cells in CD133+CDCP1+ cells, as well as in CD133+CDCP1– and CD133– subsets, was quantified throughout the label and chase phases (*n* = 3 per time point). Values shown are mean ± SD. A two-tailed unpaired *t* test was used to compare experimental groups. **p < 0.05. Bar: 50 μm. Underlying data are available in S1 Data. CD, cluster of differentiation; CDCP1, CUB-domain–containing protein 1; CidU, 5-Chloro-2′-deoxyuridine; CRC, colorectal carcinoma; CSC, cancer stem cell; EdU, 5-ethynyl-2′-deoxyuridine; FCT, *Fabpl*^*4X@132*^*-rtTA-3×(IRES-Apc*.*3374)*:*tet-Kras*^*G12D*^:*CDCP1-creERT2*:*Rosa26 CAG-loxP-stop-loxP-tdTomato*; FOLFOX, folinic acid + fluorouracil + oxaliplatin; GFP, green fluorescent protein; IdU, 5-Iodo-2′-deoxyuridine; Ki67, marker of proliferation Ki-67; *Kras*, Kirsten rat sarcoma viral oncogene homolog; MCM2, minichromosome maintenance complex component 2; mu*Kras*, mutant *Kras*; NOG, nonobese diabetic (NOD)/Shi-scid Il2rg^null^; PCNA, proliferating cell nuclear antigen; td, tandem dimer; wt*Kras*, wild-type *Kras*.

To demonstrate whether CDCP1+ CSCs represent the real culprit behind recurrence in mu*Kras* CRCs, we crossbred mice carrying tet-*Kras*^*G12D*^ with *Fabpl*^–*4X@132*^*-rtTA-3×(IRES-Apc*.*3374)* mice to enable colon-specific and doxycycline (Dox)-controlled expression of oncogenic *Kras*^*G12D*^ (S2A, S2B and S2C Fig) in the context of Apc silencing (S2D Fig) [16]. These offspring were mated with *CDCP1-creERT2* and *Rosa26 CAG-loxP-stop-loxP-tdTomato* mice to generate *Fabpl*^*4X@132*^*-rtTA-3×(IRES-Apc*.*3374)*:*tet-Kras*^*G12D*^:*CDCP1-creERT2*:*Rosa26 CAG-loxP-stop-loxP-tdTomato* (FCT) mice. FCT mice developed tubular CRCs following 14 weeks of Dox induction (S2E Fig). Colonoscopic monitoring showed reduced tumor burdens upon administration of intraperitoneal (IP) FOLFOX for 2 weeks (regression phase); relapse was detected in 11 out of 17 mice within 10–12 weeks after treatment cessation (relapse phase; S2E Fig). To perform lineage tracing on CDCP1+ CSCs, tumor-bearing FCT mice were injected with tamoxifen (Fig 2A) to specifically mark CDCP1+ cells with tdTomato (S2F Fig). Upon FOLFOX administration and withdrawal, the lineage trace experiment showed that the frequency of marked CDCP1+ cells remained constant throughout the tracing period (Fig 2B), indicating that this population is self-renewing. In contrast, the percentage of tdTomato+ tumor cells increased progressively despite decreased tumor burden in the regression phase; at week 7 after drug withdrawal, nearly 90% of relapsed tumor cells were tdTomato+ (Fig 2C and 2D). Such a percentage was significantly higher than that in untreated control tumors (S2G Fig). To summarize, CDCP1+ cells are capable of self-renewing and differentiating into the fast-dividing cells to form relapsed tumors.

Next, we exposed NOG mice carrying patient-derived orthotopic xenograft (CRC101 with *Kras*^*G13D*^) to vehicle or FOLFOX regimen for 2 weeks and followed this with CidU long-term tracing to label the cell-cycle activity of CDCP1+ subpopulation (Fig 2E). As compared with the vehicle group, the number of CDCP1+/CidU+ cells labeled by IdU (given 2 h prior to analysis) was remarkably increased in the FOLFOX group (Fig 2F), confirming the entry of CDCP1+ CSCs into the cell cycle. This further implied that CDCP1+ CSCs are the cell of origin of relapsed tumors after chemotherapy.

### CDCP1+ CSCs in mu*Kras* CRCs define a quiescent subpopulation

Similar to normal stem cells, CSCs are quiescent, slow-cycling cells endowed with long-term repopulation ability [17]. As compared with CDCP1– cells from mu*Kras* tumor cells (DKs5 and HK2-10), the matched CDCP1+ fraction expressed lower levels of markers for cell proliferation, including Ki67, PCNA, and MCM2 (Fig 2G). CDCP1+ cells were identified exclusively within the CD133+ fraction [7]. Analysis of mu*Kras* tumor cells using Hoechst plus pyronin Y staining showed that >90% of the CD133+ CDCP1+ fraction was in the G0 phase, whereas most CD133+ CDCP1– and CD133– fractions were actively cell cycling (<47% in the G0 phase). In contrast, cells were highly proliferative regardless of CDCP1 status in wt*Kras* tumor cells (Fig 2H).

We also used a chronic thymidine analog label-chase experiment to define tumor-proliferative dynamics. NOG mice carrying CRC102 or CRC108 orthotopic xenografts received 5-ethynyl-2′-deoxyuridine (EdU) through drinking water for 8 days and were killed at varying time points during a 40-day chase (Fig 2I). The label-chase experiment showed that in both CRC102 and CRC108 xenografts, CD133– cells and CD133+ CDCP1– cells rapidly acquired and diluted the EdU label, confirming a high degree of cell proliferation and turnover. However, the CD133+ CDCP1+ fraction in CRC108 xenografts, but not CRC102 xenografts, acquired EdU more slowly, labeled to a lesser extent, and maintained the label throughout the chase, suggesting a state of quiescence (Fig 2J).

### Intermediate metabolites are rerouted from glycolysis to PPP in CDCP1+ CSCs from mu*Kras* CRCs

**Fig 3:**
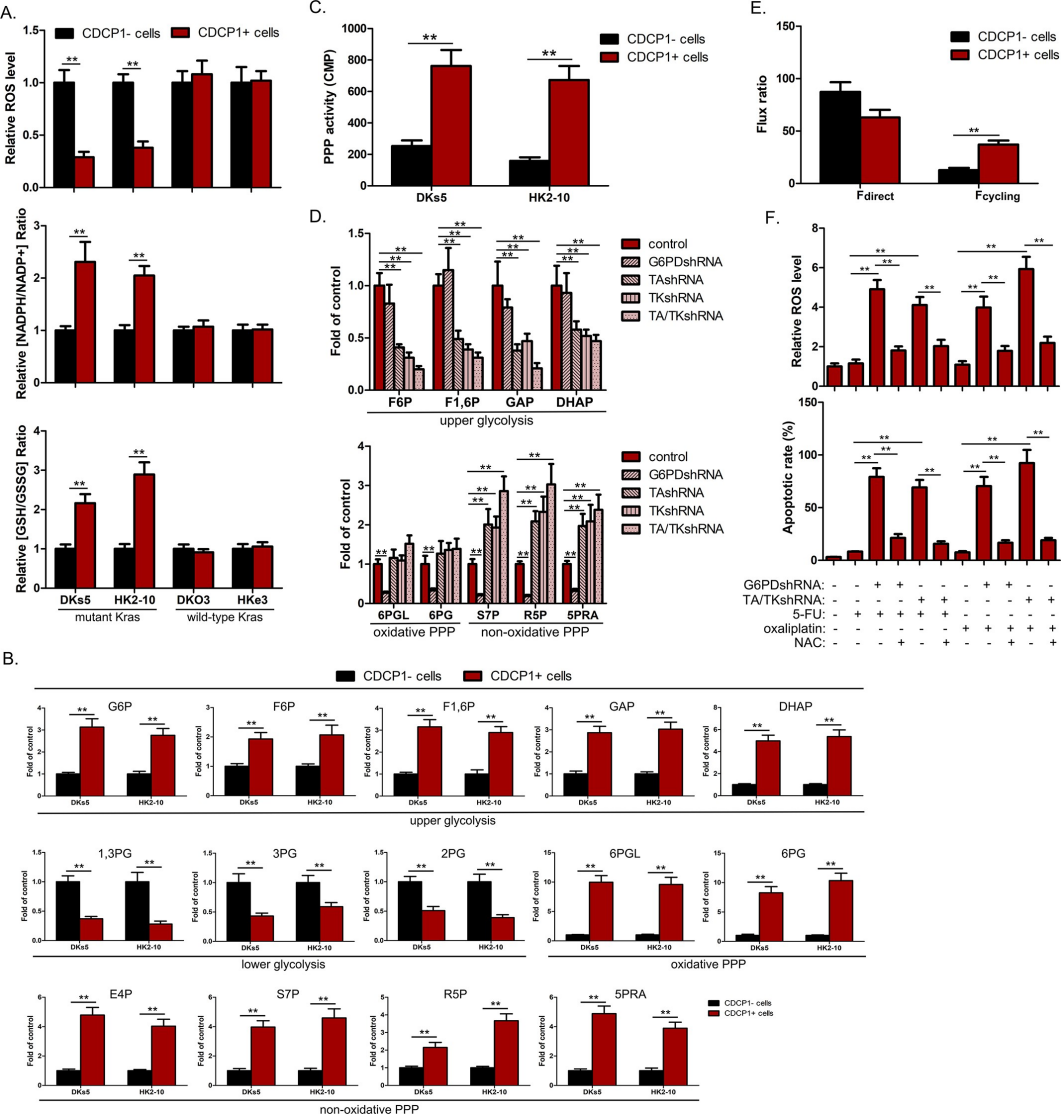
Oxidative PPP is activated in CDCP1+ CSCs from mu*Kras* CRCs. (A) ROS levels and the ratios of [NADPH/NAPD+], [GSH/GSSG] were compared between CDCP1+ and CDCP1– fractions from mu*Kras* and wt*Kras* CRC cell lines. (B) Relative intracellular metabolite levels in DKs5-derived CDCP1+ and CDCP1– cells. (C) PPP activity of CDCP1+ and CDCP1– fractions isolated from DKs5 and HK2-10 cells. PPP activity was calculated as the difference between the rate of [1-^14^C]-glucose and [6-^14^C]-glucose oxidation to [^14^C]-CO_2_ (*n* = 3). (D) Relative intracellular metabolite levels in DKs5-derived CDCP1+ cells transfected with control shRNA or shRNA targeting G6PD, TK, TA, or both TK and TA. (E) Ratio of fluxes entering directly from taken up glucose into oxidative PPP (f_direct_) or after one or multiple cycles through oxidative PPP and entering back into oxidative PPP via upper glycolysis (f_cycling_). The ratios were calculated using the m + 0 fraction of 6PG in HK2-10–derived CDCP1+ and CDCP1– cells cultured in media supplemented with [1-^13^C]-glucose. (F) HK2-10–derived CDCP1+ cells with G6PD or TK/TA KD were treated with or without antioxidant agent NAC (1 mM), together with or without 5-FU (5 μM) or oxaliplatin (1 μM). ROS levels (upper panel) and apoptotic rate (lower panel) were measured at 24 h and 72 h after treatment, respectively. All experiments were independently repeated three times in triplicate. Values shown are mean ± SD. A two-tailed unpaired *t* test was used to compare experimental groups. **p < 0.05. Underlying data are available in S1 Data. CDCP1, CUB-domain–containing protein 1; CRC, colorectal carcinoma; CSC, cancer stem cell; DHAP, dihydroxyacetone phosphate; E4P, erythrose 4-phosphate; GAP, glyceraldehyde 3-phosphate; GSH, glutathione; GSSG, glutathione disulfide; G6PD, glucose-6-phosphate dehydrogenase; KD, knockdown; *Kras*, Kirsten rat sarcoma viral oncogene homolog; mu*Kras*, mutant *Kras*; NAC, N-acetylcysteine; PPP, pentose phosphate pathway; ROS, reactive oxygen species; R5P, ribose 5-phosphate; shRNA, short hairpin RNA; S7P, sedoheptulose 7-phosphate; TA, transaldolase; TK, transketolase; wt*Kras*, wild-type *Kras*; 1,3PG, 1,3-bisphosphoglycerate; 2/3PG, 2/3-phosphoglycerate; 5PRA, 5-phosphoribosylamine; 5-FU, 5-fluoropyrimidine; 6PG, 6-phosphogluconate; 6PGL, 6-phosphoglucono-D-lactone.

It is believed that quiescent CSCs keep lower ROS level to help them survive from chemotherapy-induced ROS [18]. As compared to the CDCP1– fraction in mu*Kras* tumor cells (DKs5 and HK2-10), CDCP1+ CSCs exhibited relatively lower levels of intracellular ROS, accompanied with remarkably higher levels of the [NADPH/NADP+] and [glutathione/glutathione disulfide] ([GSH/GSSG]) ratios (Fig 3A). However, the difference of the redox parameters between CDCP1– and CDCP1+ fractions was negligible in wt*Kras* tumor cells (DKO3 and HKe3) (Fig 3A). GSH redox status is dependent on NADPH production from various respiratory substrates. To determine which NADPH-producing enzyme(s) may contribute to the observed redox state in CDCP1+ CSCs from mu*Kras* CRCs, we knocked down NADPH-producing enzymes, including glucose-6-phosphate dehydrogenase (G6PD), 6-phosphogluconate dehydrogenase (6PGD), malic enzyme 1 (ME1), methylenetetrahydrofolate dehydrogenase 1 and 2 (MTHFD1/2), and isocitrate dehydrogenase 1 and 2 (IDH1/2) in DKs5-derived CDCP1+ cells. The ratio of [NADPH/NADP+] was reduced by 77% and 69% in G6PD knockdown (KD) and 6PGD KD cells, respectively, while KD of ME1, MTHFD1/2 or IDH1/2 did not exert such apparent effects on redox balance (S3A Fig). These data suggested that NADPH is mainly produced through the pentose phosphate pathway (PPP). The PPP is a metabolic pathway parallel to glycolysis. In glycolysis hexose phosphates, hexose bisphosphates, and glyceraldehyde 3-phosphate (GAP)/dihydroxyacetone phosphate (DHAP) were elevated in CDCP1+ cells from mu*Kras* tumors compared to the matched CDCP1– fraction (Fig 3B). However, concentrations of 2/3-phosphoglycerate (xPG) and phosphoenolpyruvate (PEP), metabolites of the lower glycolysis, were significantly decreased (Fig 3B). The strongest difference was observed for 6-phosphoglucono-D-lactone (6PGL) and 6-phosphogluconate (6PG) in the oxidative branch of the PPP (Fig 3B). The levels of metabolites of the nonoxidative branch of PPP (i.e., erythrose 4-phosphate [E4P], sedoheptulose 7-phosphate [S7P], and pentose phosphates) showed an attenuated increase in CDCP1+ CSCs (Fig 3B). The increase in PPP metabolites was paralleled by an accumulation of the purine precursor 5-phosphoribosylamine (5PRA) (Fig 3B), suggesting an increased purine de novo biosynthesis possibly needed for fast and efficient DNA repair mechanisms in CSCs.

Next, we attempted to corroborate our findings using ^14^C-glucose. Increased flux through the oxidative PPP pathway, as measured by the amount of released ^14^CO_2_ from [1-^14^C]-glucose, was indeed observed in CDCP1+ cells (Fig 3C). This result was further confirmed by metabolically labeling the cells with [1,2-^13^C]-glucose and measuring the relative isotopic enrichment of doubly versus singly [^13^C]-labeled lactate, pyruvate, and 3-phosphoglycerate by liquid chromatograph-triple quadrupole mass spectrometry (LC-MS) analysis, an established method that directly tracks labeled glucose flux simultaneously through glycolysis and the PPP [19]. Specifically, at least 2.6-fold increase in PPP flux was observed in CDCP1+ cells as compared to the matched CDCP1– fraction (S3B Fig).

To investigate the detailed rerouting and infer the directionality of fluxes in oxidative and nonoxidative PPP, we analyzed the metabolic response after depletion of G6PD, transketolase (TK), or transaldolase (TA) in CDCP1+ CSCs. In G6PD KDs, the levels of 6PGL and 6PG were significantly attenuated, demonstrating that their accumulation is caused by activation of the oxidative PPP involving G6PD (Fig 3D). The concentrations of pentose phosphates and S7P in the G6PD KDs were also reduced (Fig 3D), suggesting that the accumulation in pentose phosphates results from an increased higher flux from 6PG but not from glycolysis via reversed TK and TA. Accordingly, single or double KDs of TK and TA led to even stronger accumulation of pentose phosphates and 5PRA pools, accompanied by a pronounced decrease of metabolites in upper glycolysis (Fig 3D). Thus, the carbon flux from the oxidative branch returns to glycolysis through TA and TK, leading to accumulation of upper glycolytic metabolites.

Upon strong cycling of carbon through oxidative and nonoxidative PPP, a fraction of carbon backbones may be converted back to G6P by the reversible phosphoglucoisomerase (PGI) and cycle again through oxidative PPP to further increase NADPH production. Exploiting the loss of the 1-C of glucose in the oxidative PPP and the measured 6PG_m+0_ fraction of the [1-^13^C] glucose experiment, we estimated the relative contribution of unlabeled hexose phosphates (f_cycling_) to the oxidative PPP to increase from 10.6% ± 5.9% in CDCP1– cells to 48.2% ± 12.6% in the matched CDCP1+ subpopulation (Fig 3E).

To verify that metabolic rerouting in the PPP is necessary for protection of CDCP1+ CSCs against oxidative stress from chemotherapy, we investigated the change in NADPH/NADP+ ratio and ROS levels with perturbed PPP functionality. KDs of either G6PD or TK/TA in CDCP1+ cells resulted in higher ROS level and lower NADPH/NADP+ ratio (S3C Fig). Stress conditions induced by 5-FU or oxaliplatin resulted in further elevated ROS levels (Fig 3F, upper) and subsequent apoptosis in either G6PD or TK/TA KDs (Fig 3F, lower). Treatment with antioxidant N-acetylcysteine (NAC) in either G6PD or TK/TA KDs rescued the increased ROS and drug susceptibility (Fig 3F).

### Sirt5 demalonylates TPI K56 to suppress its enzyme activity

**Fig 4:**
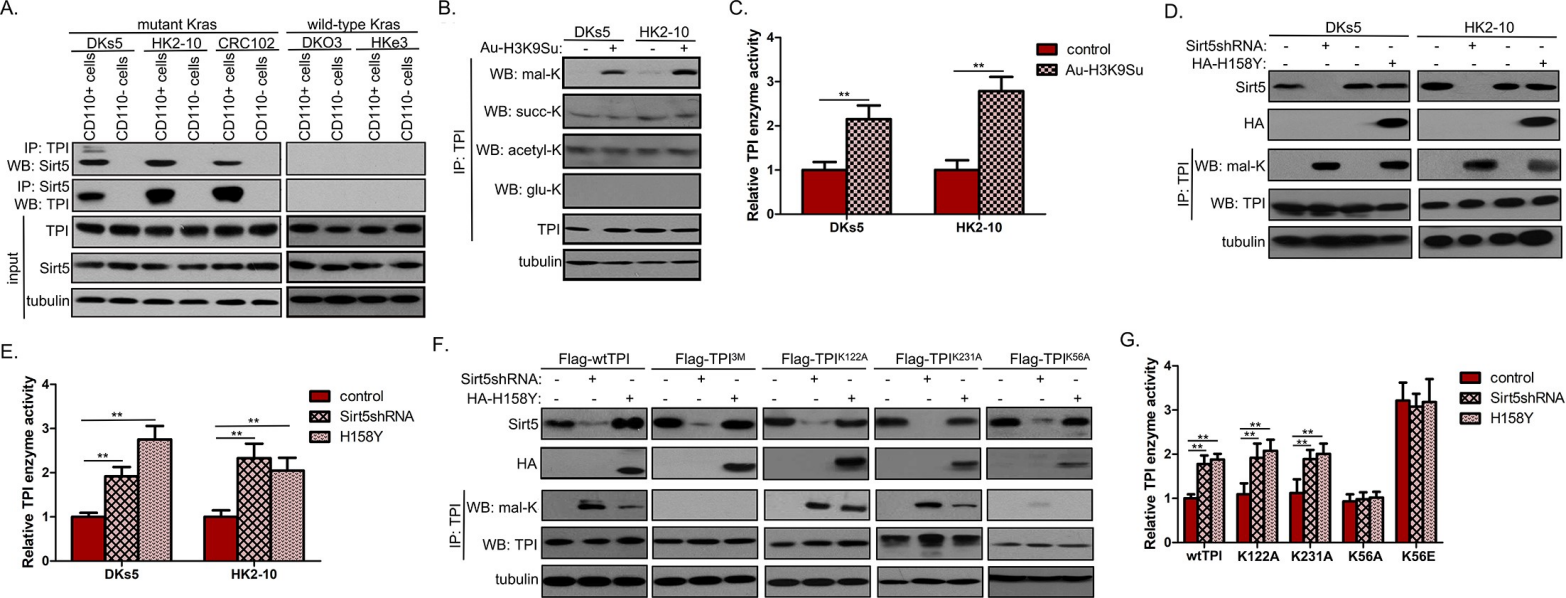
Sirt5 promotes TPI demalonylation to suppress its enzyme activity. (A) Co-IP of Sirt5 and TPI in CDCP1– and CDCP1+ fractions from mu*Kras* and wt*Kras* CRC cells. (B–C) DKs5 and HK2-10–derived CDCP1+ cells were treated with Au-H3K9Su for 48 h, after which lysine modifications (B) and enzyme activity (C) of endogenous TPI were determined. (D–E) DKs5 and HK2-10–derived CDCP1+ cells were transfected with Sirt5 shRNA or HA-H158Y mutant, after which lysine malonylation (D) and enzyme activity (E) of endogenous TPI were determined. (F–G) The indicated Flag-tagged TPI vectors were transfected into DKs5-derived CDCP1+ cells with stable Sirt5 KD or H158Y overexpression. WT and mutant TPI proteins were purified by Flag beads and eluted with Flag peptide, followed by determination of their lysine malonylation (F) and enzyme activity (G). All experiments were independently repeated three times in triplicate. Values shown are mean ± SD. A two-tailed unpaired *t* test was used to compare experimental groups. **p < 0.05. Underlying data are available in S1 Data. acetyl-K, lysine acetylation; CD, cluster of differentiation; CDCP1, CUB-domain–containing protein 1; CRC, colorectal carcinoma; glu-K, lysine glutarylation; HA, hemagglutinin; IP, immunoprecipitation; KD, knockdown; *Kras*, Kirsten rat sarcoma viral oncogene homolog; mal-K, lysine malonylation; mu*Kras*, mutant *Kras*; shRNA, short hairpin RNA; Sirt5, silent mating type information regulation 2 homolog 5; succ-K, lysine succinylation; TPI, triosephosphate isomerase; WB, western blot; WT, wild type; wt*Kras*, wild-type *Kras*.

The decreased concentration of xPG and PEP suggested that activities of the enzymes involved in the lower glycolysis is blocked, which can lead to an accumulation of upper glycolytic metabolites and thereby induces an increased flux into PPP through both the oxidative and nonoxidative branches. Metabolic enzyme activity is controlled by three principle levels: the amount of enzyme, the catalytic activity, and the accessibility of substrates. Of note, among the enzymes of the lower glycolysis (TPI; glyceraldehyde-3-phosphate dehydrogenase [GAPDH], phosphoglycerate kinase [PGK], phosphoglucomutase [PGM], enolase, and pyruvate kinase [PK]), only activity of endogenous TPI was suppressed in CDCP1+ cells from mu*Kras* CRCs as compared to the matched CDCP1– fraction (S4A Fig). No difference in the protein expression levels of TPI was observed (S4B Fig), indicating that the decrease in activity was not due to reduced TPI expression. To clarify the regulatory mechanism(s) underlying TPI activity, we performed coimmunoprecipitation experiments followed by mass spectrometry (MS) analysis in CRC102-derived CDCP1+ cells. This screen revealed that the most common interaction partner of TPI corresponds to Sirt5 (S2 Table). Sirt5 coprecipitated with TPI in CDCP1+ cells from mu*Kras* CRCs, while the association was lost in the CDCP1– counterpart. Such an interaction was also not observed in wt*Kras* tumors (Fig 4A). Sirt5 is a global regulator of lysine acetylation, succinylation, malonylation, and glutarylation in mitochondria [20–23]. Upon Au-H3K9Su (gold nanoparticles tethered with H3K9Su, a selective Sirt5 inhibitor) treatment, the malonylation level of TPI was increased (Fig 4B), accompanied by significantly increased TPI enzyme activity (Fig 4C). TPI demalonylation was only detectable in CDCP1+ CSCs from mu*Kras* CRCs (S4C Fig), suggesting that demalonylation negatively regulates TP1 activity. Besides, TPI malonylation was remarkably increased by Sirt5 KD in CDCP1+ cells (Fig 4D), with concomitant activation in catalytic activity by as much as >2-fold (Fig 4E). Overexpression of a catalytically dead Sirt5 mutant (H158Y), which still strongly interacted with endogenous TPI (S4D Fig), had a similar effect on TPI malonylation and its enzyme activity (Fig 4D and 4E). These data indicated that Sirt5 regulates TPI activity by controlling its malonylation.

To identify Sirt5-targeted demalonylation site(s) of TPI, we overexpressed Flag-tagged TPI in CRC108-derived CDCP1+ cells. Tandem MS analysis identified three lysine malonylation sites (K56, K122, and K231) upon Sirt5 KD (S4E Fig). We constructed a malonylation-resistant TPI mutant (TPI 3M) in which all three lysines were mutated to alanines (A). Immunoblotting analyses showed that TPI 3M mutation abrogated malonylation of TPI in Sirt5-depleted CDCP1+ cells (Fig 4F). We further constructed single-site TPI mutants, including TPI K56A, K122A, and K231A. Among them, TPI K122A and K231A greatly increased TPI malonylation in Sirt5-depleted CDCP1+ cells (Fig 4F). Unlike wild-type (WT) TPI and K122A/K231A mutants, the K56A mutant displayed a negligible response to either Sirt5 KD or Sirt5 H158Y overexpression in changing malonylation level (Fig 4F), as well as enzyme activity (Fig 4G). We also generated TPI KD CDCP1+ cells and transfected these cells with the K56E mutant (S4F Fig) (henceforth referred to as TPI^K56E/KD^ cells), in which K56 of TPI was substituted with glutamic acid to mimic constitutive malonylation. The K56E mutant exhibited constitutive enzyme activity regardless of Sirt5 or Sirt5 H158Y expression status (Fig 4G). Together, these data suggested that indicating that K56 in TPI is the key malonylation site directly targeted by Sirt5.

### TPI^K56^ demalonylation induces metabolic flux through PPP in CDCP1+ CSCs to mediate postchemotherapeutic recurrence

**Fig 5:**
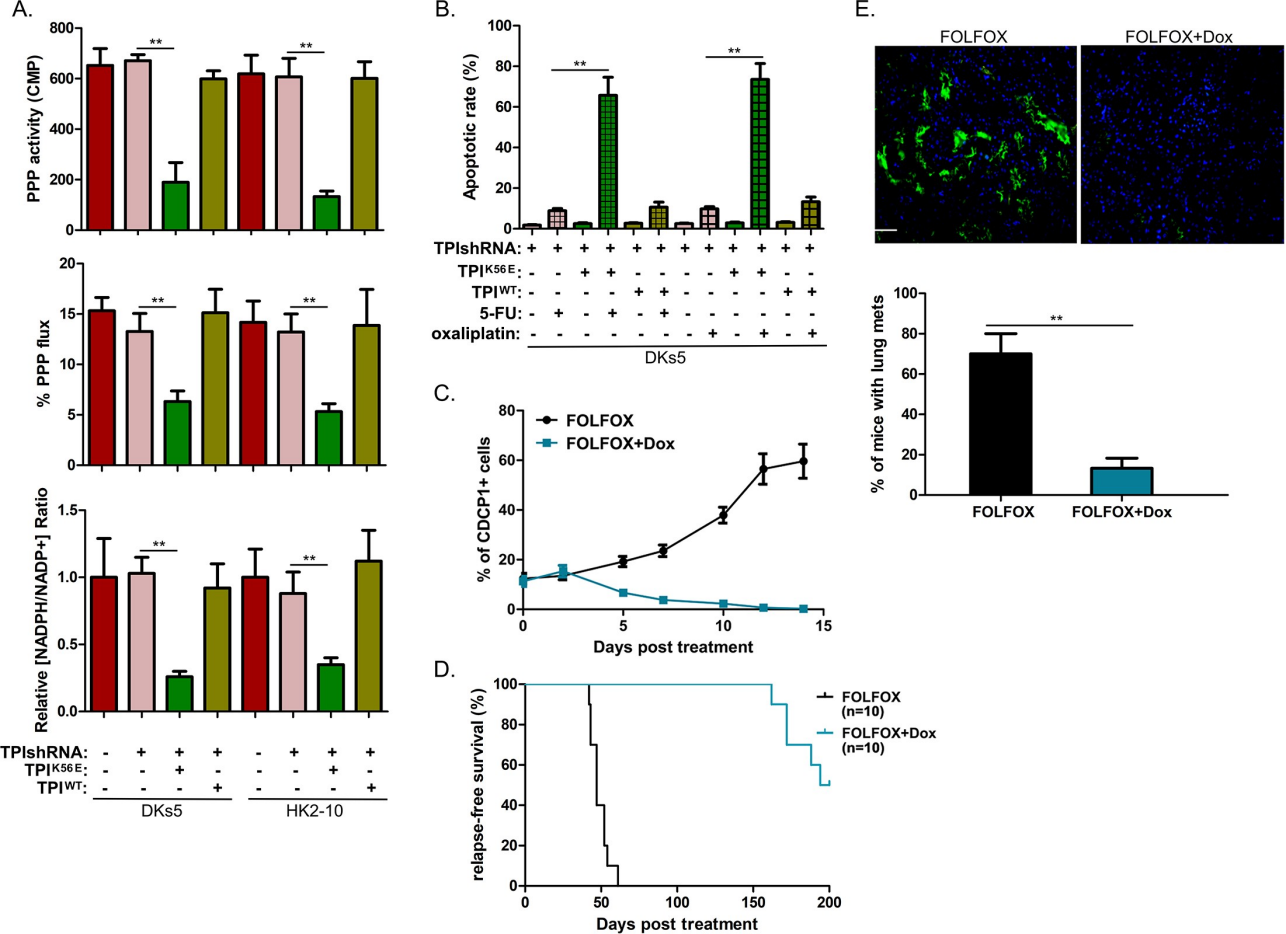
TPI demalonylation at K56 decreases PPP flux in CDCP1+ CSCs and is indispensable for postchemotherapeutic recurrence. (A) The WT TPI or TPI^K56E^ vector was transfected into DKs5 and HK2-10–derived CDCP1+ cells with stable TPI KD, after which PPP activity, percentage of PPP flux, and the ratio of [NADPH/NADP+] were determined. (B) HK2-10–derived CDCP1+ cells with TPI KD were transfected with the WT TPI or TPI^K56E^ vector, together with or without 5-FU (5 μM) or oxaliplatin (1 μM) treatment. The apoptotic rate was measured at 72 h after treatment. (C) The frequency of CDCP1+ cells between FOLFOX and FOLFOX + Dox groups in S5D Fig was determined by flow cytometry (*n* = 3 per time point). (D) RFS was compared between FOLFOX and FOLFOX + Dox groups in S5D Fig by Kaplan–Meier survival analyses. (E) Upper panels: representative lung sections from mice with recurrent tumors in S5D Fig showing GFP-positive lung metastases; lower panel: percentage of mice with visible lung metastases (*n* = 10 per group). *P*-value is determined by the log-rank test. Sample size is indicated in parentheses. Throughout, values shown are mean ± SD. *P*-values were calculated by two-tailed *t* test unless otherwise indicated. **p < 0.05. Underlying data are available in S1 Data. CDCP1, CUB-domain–containing protein 1; CSC, cancer stem cell; Dox, doxycycline; FOLFOX, folinic acid + fluorouracil + oxaliplatin; GFP, green fluorescent protein; KD, knockdown; PPP, pentose phosphate pathway; shRNA, short hairpin RNA; TPI, triosephosphate isomerase; WT, wild type; 5-FU, 5-fluoropyrimidine.

To test whether glucose flux through the PPP requires TPI^K56^ demalonylation, we cultured TPI-depleted CDCP1+ cells rescued with Flag-TPI WT or K56E (S4F Fig). TPI^K56E/KD^ cells displayed decreased oxidative PPP activity, as determined by the amount of released ^14^CO_2_ from [1-^14^C]-glucose. In contrast, PPP activity remained unaffected in TPI^WT/KD^ cells (Fig 5A, upper). The observed decrease in PPP activity for TPI^K56E/KD^ cells compared to TPI^WT/KD^ cells was likely due to the stimulating effects of the K56E mutation on TPI activity (Fig 4G). Exploiting the relative isotopic enrichment of doubly versus singly [^13^C]-labeled lactate, pyruvate, and 3-phosphoglycerate in the [1,2-^13^C] glucose experiment, we found that TPI^K56E/KD^ cells showed a 2.1-fold reduction in PPP flux, whereas TPI^WT/KD^ cells showed no such change (Fig 5A, middle). As a consequence, TPI^K56E/KD^ cells displayed a remarkable reduction in the [NADPH/NADP+] ratio (Fig 5A, lower) and were more sensitive to 5-FU, as well as oxaliplatin, as compared to TPI^KD^ cells (Fig 5B), whereas TPI^WT/KD^ cells did not exhibit similar results (Fig 5A and 5B). Together, TPI inactivation caused by demalonylation at K56 is the key determinant for reorganization of PPP flux in CDCP1+ CSCs.

To determine the significance of TPI^K56^ demalonylation in CDCP1+-CSC–mediated postchemotherapeutic recurrence, we infected CRC108 cells with two retroviral vectors in which hemagglutinin (HA)-TPI^K56E^ and TPI shRNA were expressed under the dual control of the CDCP1-responsive promoter and the Dox-inducible activator rtTA (S5A Fig). Dox treatment induced selective depletion of endogenous TPI and expression of HA-TPI^K56E^ in the CDCP1+ fraction (S5B Fig). Green fluorescent protein (GFP)-labeled CRC108 cells overexpressing these vectors were orthotopically transplanted into NOG mice and were allowed to grow until the tumor reached a comparable size prior to receiving FOLFOX regimen with or without Dox. Dox treatment alone failed to eradicate CDCP1+ cells (S5C Fig) and therefore had no impact on tumor growth (S5D Fig). The pool of CDCP1+ CSCs in TPI^K56E/KD^ tumors was exhausted within 14 days of combined FOLFOX and Dox treatment (Fig 5C). FOLFOX plus Dox postponed the time to recurrence (by >8 weeks versus mice receiving FOLFOX alone; Fig 5D) and decreased the number of metastatic pulmonary lesion (Fig 5E).

### K56 demalonylation impairs the formation of dimeric TPI to inhibit its enzyme activity

**Fig 6:**
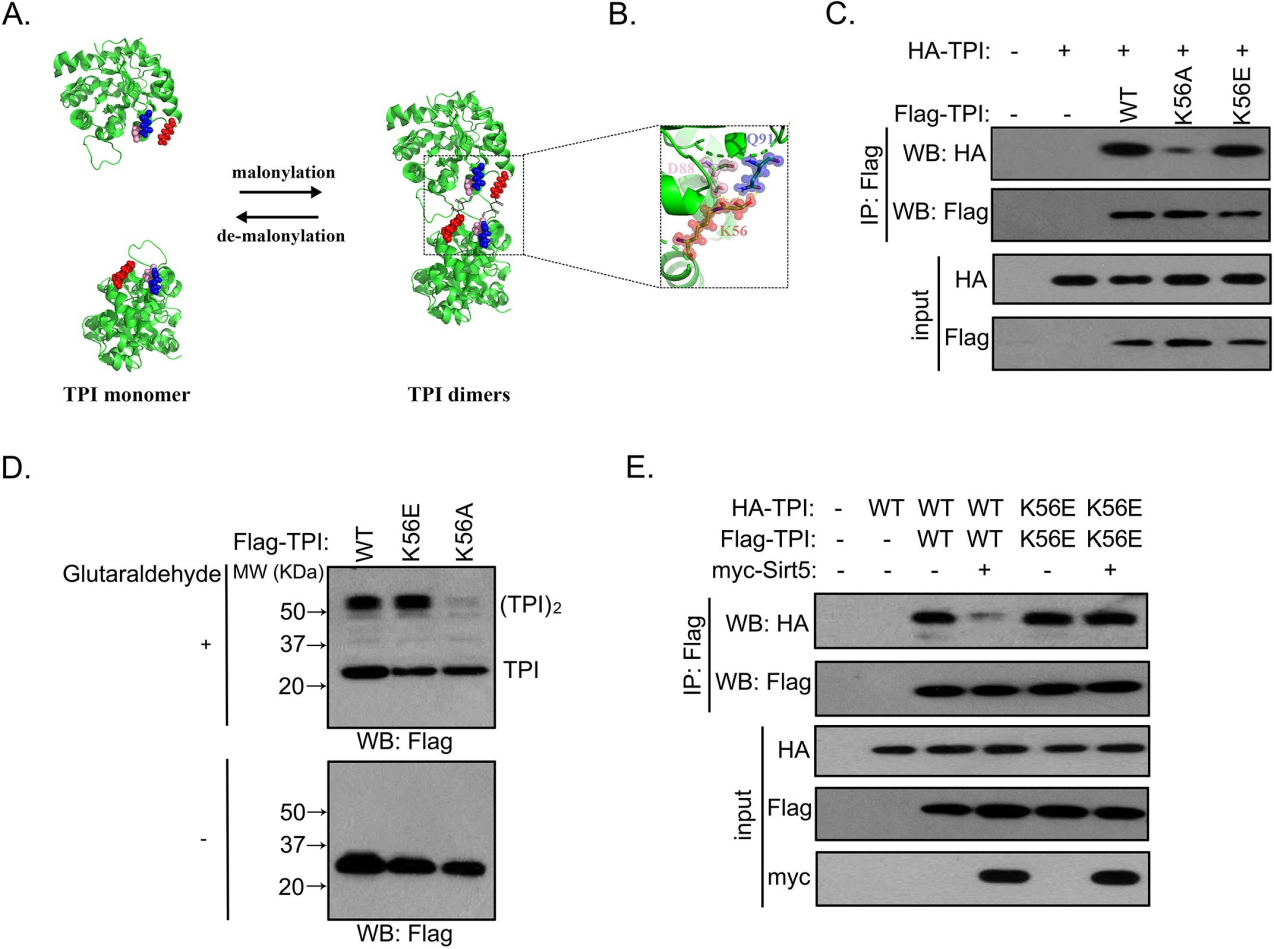
K56 demalonylation impairs the formation of dimeric TPI and inhibits its enzyme activity. (A) Malonylation of K56 (red) induces the formation of hydrogen bonds with D88 (pink) and Q91 (blue), stabilizing the dimerization of TPI. (B) Close-up view of the interactions at the TPI dimer interface with K56 (Protein Data Bank: 4POC). Four out of the eight contacts dependent on K56 malonylation are indicated with a dashed line. The other four hydrogen bonds are the equivalent companions on the reverse side of the protein and are not visible in the picture. (C) Flag-tagged TPI or K56A/K56E mutants were each expressed in HK2-10–derived CDCP1+ cells coexpressing HA-tagged TPI. The interaction between Flag-tagged and HA-tagged proteins was determined by western blot. (D) Flag-tagged TPI or K56A/K56E mutants were each expressed in HEK293T cells, followed by treatments with or without 0.025% glutaraldehyde. The formation of TPI monomer and dimer was determined by western blot. (E) Flag-tagged TPI or K56E mutant was each expressed in HEK293T cells coexpressing HA-tagged TPI or K56E mutant, respectively. Cells were then transfected with or without myc-Sirt5, and the interaction between Flag-tagged and HA-tagged proteins was determined by western blot. CDCP1, CUB-domain–containing protein 1; HA, hemagglutinin; HEK, human embryonic kidney; IP, immunoprecipitation; MW, molecular weight; Sirt5, silent mating type information regulation 2 homolog 5; TPI, triosephosphate isomerase; WB, western blot; WT, wild type.

The TPI enzyme exists as a mixture of monomer and dimer, but only the dimeric form is catalytically active [24]. The crystal structure of the TPI dimer showed that K56 is localized at the interface (Protein Data Bank: 4POC). Malonylation of K56 promotes the formation of hydrogen bonds at the dimer interface with D88 and Q91 and thereby stabilizes the dimerization of TPI (Fig 6A and 6B). Next, we attempted to test whether K56 demalonylation inhibits TPI activity through impairing the integrity of dimer interface and subsequently the formation of active dimers of TPI. To test this hypothesis, we determined the interaction between two differentially tagged TPI proteins, HA-TPI and Flag-TPI, in CDCP1+ cells. Strikingly, substitution of K56A, but not K56E, primarily disrupted the interaction between TPI subunits (Fig 6C). A glutaraldehyde cross-linking assay showed that the K56A mutant displayed impaired ability to form dimers when compared to WT TPI or the K56E mutant (Fig 6D), further suggesting that K56 demalonylation largely hinders the interaction between TPI subunits. Moreover, Sirt5 overexpression decreased the binding by approximately 85% between the two differentially tagged proteins of WT TPI but exerted no impact on the interaction between Flag-tagged and HA-tagged K56E mutants (Fig 6E).

### CDCP1 cleavage is essential for the Sirt5–TPI interaction in CDCP1+ CSCs from mu*Kras* CRCs

**Fig 7:**
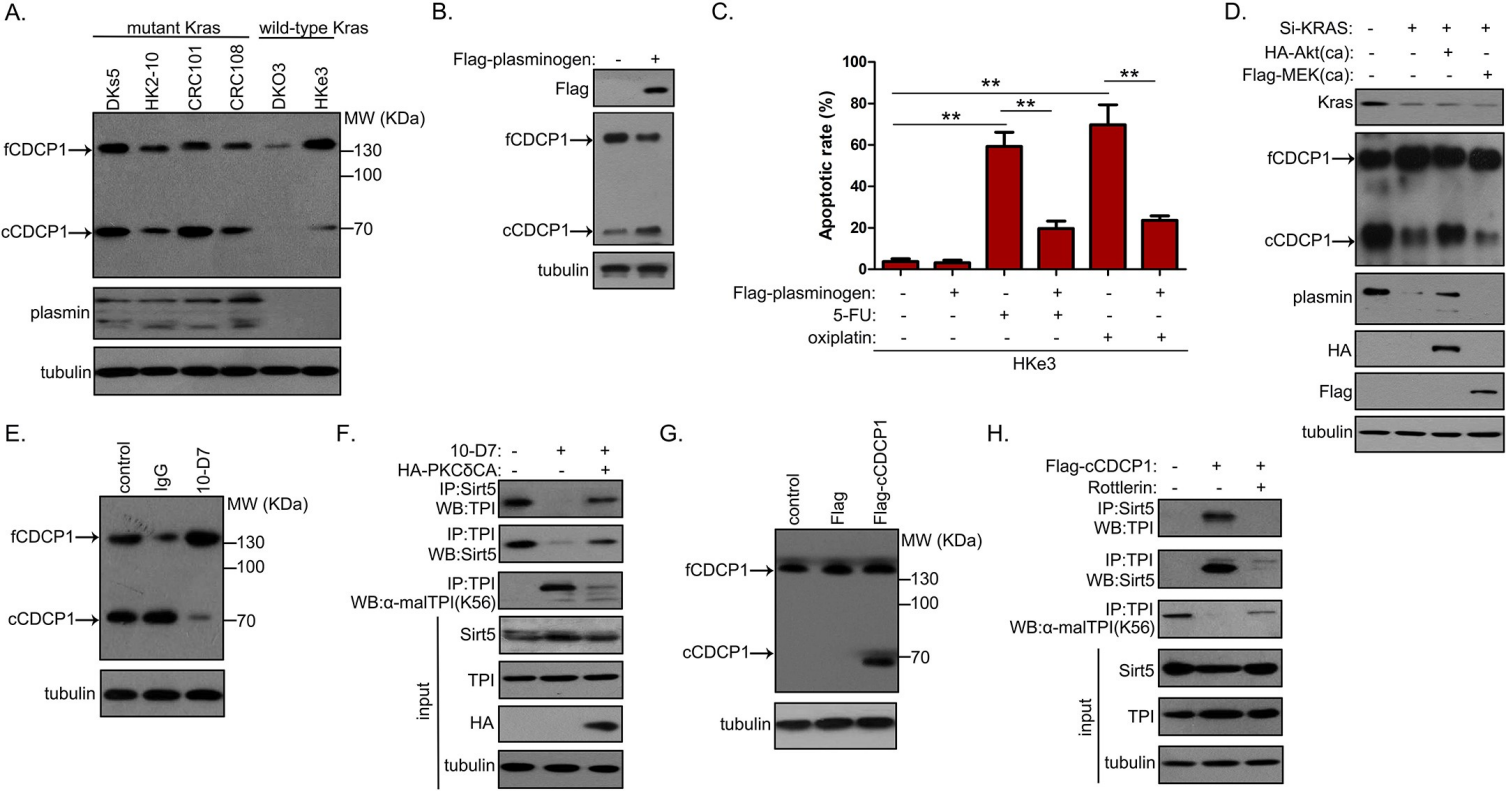
Cleaved CDCP1 in mu*Kras* CRCs promotes Sirt5–TPI association to stimulate TPI^K56^ demalonylation. (A) Western blot analysis of CDCP1 cleavage and plasmin secretion in mu*Kras* and wt*Kras* CRCs. (B) Western blot analysis of CDCP1 cleavage in HKe3-derived CDCP1+ cells overexpressing Flag plasminogen. (C) HKe3-10–derived CDCP1+ cells were transfected with the Flag-plasminogen vector, together with or without 5-FU (5 μM) or oxaliplatin (1 μM) treatment. The apoptotic rate was measured at 48 h after drug treatment. (D) CRC108-derived CDCP1+ cells were transfected with siRNA targeting *Kras*, together with or without the HA-AktCA or Flag-MEKCA mutant, following which CDCP1 cleavage and plasmin expression were analyzed by western blot. (E) Analysis of CDCP1 cleavage in CRC108-derived CDCP1+ cells pretreated with 20 μg/ml CDCP1 mAb 10-D7 or control IgG for 24 h. (F) CRC108-derived CDCP1+ cells were pretreated with 20 μg/ml 10-D7 and/or HA-PKCδCA mutant for 48 h prior to assessment of Sirt5–TPI association and TPI K56 malonylation. (G) cCDCP1 expression in CRC108-derived CDCP1– cells overexpressing Flag-cCDCP1. (H) CRC108-derived CDCP1− cells transfected with Flag-cCDCP1 were treated with or without rottlerin (2.5 μM) for 48 h prior to assessment of Sirt5–TPI association and TPI K56 malonylation. Underlying data are available in S1 Data. Akt, RAC-alpha serine/threonine-protein kinase; AktCA, catalytically active mutant of Akt; cCDCP1, cleaved CDCP1; CDCP1, CUB-domain–containing protein 1; CRC, colorectal carcinoma; fCDCP1, full-length CDCP1; HA, hemagglutinin; IgG, immunoglobulin G; IP, immunoprecipitation; *Kras*, Kirsten rat sarcoma viral oncogene homolog; mAb, monoclonal antibody; malTPI, malonylated TPI; MEK, mitogen-activated protein kinase; MEKCA, catalytically active mutant of MEK; mu*Kras*, mutant *Kras*; MW, molecular weight; PKCδ, protein kinase C-delta; PKCδCA, catalytically active mutant of PKCδ; Si-KRAS, KRAS siRNA; siRNA, small interfering RNA; Sirt5, silent mating type information regulation 2 homolog 5; TPI, triosephosphate isomerase; WB, western blot; wt*Kras*, wild-type *Kras*; 5-FU, 5-fluorpyrimidine.

Finally, we investigated the potential mechanisms facilitating the Sirt5–TPI interaction in CDCP1+ CSCs. To become functionally active, the full-length CDCP1 (135 kDa) is cleaved by serine proteases to generate a membrane-retained 70 kDa fragment. The C-terminus of the resulting fragment acts as a docking platform for activation of downstream events [8]. CDCP1 existed mainly in the full-length form in wt*Kras* CRCs and in a cleaved form in mu*Kras* tumors (Fig 7A). This phenomenon could be attributed to negligible secretion of plasmin, a major serine protease responsible for CDCP1 cleavage [25], in wt*Kras* tumors (Fig 7A). Indeed, ectopic expression of plasminogen, the inactive precursor of plasmin, led to increased CDCP1 cleavage (Fig 7B) and decreased chemosensitivity (Fig 7C) in HKe3-derived CDCP1+ cells. The consistently higher plasmin expression in mu*Kras* CRCs compared with wt*Kras* tumors points to a correlation between *Kras* activation and plasmin expression. To test this, we performed mutant *Kras* KD, which markedly decreased plasmin expression and abolished CDCP1 cleavage in CRC108 cells (Fig 7D). The effects observed following mutant *Kras* KD were largely rescued by overexpression of a constitutively active Akt mutant but not a constitutively active mitogen-activated protein kinase (MEK) mutant (Fig 7D), suggesting that the phosphoinositide 3-kinase (PI3K)/RAC-alpha serine/threonine-protein kinase (Akt) pathway mediates plasmin expression downstream of *Kras*. Because TPI demalonylation was only observed in CDCP1+ CSCs from mu*Kras* tumors (S4C Fig), we asked whether CDCP1 cleavage is essential for TPI^K56^ malonylation. We generated and verified an antibody specifically against malonylated TPI^K56^ (anti-TPI malonyl-K56) (S6A, S6B and S6C Fig). Treatment of 10-D7 (a monoantibody that prevents plasmin-induced CDCP1 cleavage [25]; Fig 7E) in CDCP1+ cells treatment disrupted the Sirt5–TPI complex and abrogated TPI^K56^ demalonylation (Fig 7F). Because protein kinase C-delta (PKCδ) signaling lies downstream of the cleaved CDCP1, the effects of 10-D7 treatment in CDCP1+ cells were greatly rescued by constitutively activated PKCδ (Fig 7F). On the contrary, we overexpressed cleaved CDCP1 (cCDCP1) in the matched CDCP1– cells (Fig 7G) and cocultured these cells with rottlerin (a selective inhibitor of PKCδ). Immunoblotting analyses showed that cCDCP1 promoted the association between Sirt5 and TPI and induced TPI demalonylation at K56, both of which were completely blocked by rottlerin (Fig 7H).

## Discussion

Most tumor therapies, including chemo- or targeted therapies and ionizing radiation, preferentially affect cycling cells but spare quiescent cells. Activating mutations of the prototypical oncogene *Kras* drives cell-cycle progression in CRCs [26]. We showed that despite exhibiting *Kras* activity, functionally defined CDCP1+ CSCs in mu*Kras* CRCs are characterized as quiescent and do not respond to chemotherapeutic agents. Drug sensitivity is, therefore, a functional property that varies between the heterogeneous cell types comprising mu*Kras* CRCs. More importantly, greater lineage traces from CDCP1+ cells following therapy suggested that this subpopulation is the unit of selection responsible for postchemotherapeutic recurrence. Because stem-cell signature expression in various tumor correlates inversely with outcome [27], we proposed that the degree of CDCP1 at the gene or protein level relates to the size of the clinically essential pool that can cause relapse. Indeed, analysis of mu*Kras* CRC samples showed higher CDCP1 expression in patients with poorer response to neoadjuvant chemotherapy and higher rate of lung recurrence.

Quiescent CSCs preferentially exhibit lower levels of intracellular ROS, which accounts for their resistance to chemotherapeutic drugs [18]. On the other hand, stringent regulation of redox status is critical to the control of stem-cell quiescence and to the maintenance of the stem-cell pool [28, 29]. Unfortunately, how CSCs control ROS production and scavenging still remains poorly understood. GSH is one of the most important antioxidants and scavenges ROS directly or as a cofactor of the glutathione and thioredoxin systems [30]. Reduced NADPH—which is produced in different biochemical pathways, including the PPP, the citric acid cycle (TCA cycle), and folate metabolism—is involved in continuously replenishing the GSH pools as a cofactor of glutathione reductase [31–33]. In the current study, analysis in CDCP1+ CSCs demonstrated that metabolic flux is rerouted from glycolysis into oxidative PPP, as indicated by an accumulation of pentose phosphate and upper glycolytic metabolites and a decrease of metabolites in lower glycolysis. Inhibition of glycolytic enzymes (e.g., GAPDH and PK M2) has been shown to lead to rerouting of glucose flux toward anabolic processes [34, 35]. Our results suggest that inactivation of TPI, which functions upstream of these enzymes and is a major classical regulatory point for central carbon flow, exerts profound control over metabolism in CDCP1+ CSCs. This inactivation maximizes the flux of sugar phosphates in the oxidative PPP, potentially through multiple cycling, and thus production of NADPH to counteract chemotherapy-induced formation of ROS. It also raises a question regarding how CDCP1+ CSCs can inactivate TPI to modulate cellular NADPH homeostasis. We discovered for the first time, to our knowledge, that TPI is a direct substrate of Sirt5. Sirt5 is a member of the NAD-dependent sirtuin family that regulates acetylome signaling networks via posttranslational modifications (PTMs) involving various lysine acylation [21–23]. PTM opens up the opportunity to redirect metabolic flux in response to specific signaling pathways. Here, the interaction between Sirt5 and TPI was attributed to the presence of cleaved CDCP1 in mu*Kras* CRCs. CDCP1-cleavage–induced signaling cascade constituted a unique mechanism whereby proteolytically cleaved CDCP1 enhances the Sirt5–TPI protein interaction and meanwhile increases the demalonylation activity of Sirt5, thereby helping CDCP1+ CSCs to maintain NADPH homeostasis through regulating TPI malonylation status in a Sirt5-dependent manner. Sirt5 has been demonstrated to regulate metabolic enzymes by PTMs and control several cellular metabolism pathways, including TCA cycle and glycolysis [21, 22], the urea cycle [36, 37], and fatty acid oxidation [20, 38]. We found that Sirt5-mediated lysine demalonylation at K56 causes enzymatic inhibition of TPI. K56 is located near the dimer interface, which is believed to be critical for the stability and integrity of the active form of TPI [39]. K56A substitution displays a significant reduction in TPI catalysis and is defective in dimer formation. Therefore, our study provides insights into the role of K56 demalonylation in coordinating the dimer-to-monomer transition of TPI. Because blocking TPI demalonylation enhances its activity and resets the cellular metabolism toward glycolysis, therapeutics that target the metabolic “Achilles heel” of CDCP1+ CSCs by inhibiting TPI demalonylation in parallel to tumor debulking by chemotherapy may be required for optimal therapeutic specificity, yielding the most durable tumor remission in mu*Kras* CRCs. Last but not least, inhibiting TPI demalonylation by Sirt5 inhibitor H3K9Su is an imperfect treatment because of its low membrane permeability. This opens up increased demands for the development of more potent and more cell-permeable inhibitors specific for Sirt5 to combat mu*Kras* CRCs.

## Materials and methods

### Ethics statement

Human CRC specimens were obtained with written informed consent from all subjects before surgery and in accordance with the ethical standards of the Institutional Review Board of Second Military Medical University (20160027). All mouse experiments were conducted in accordance with standard operating procedures approved by the Institutional Animal Care Use Committee of Second Military Medical University (protocol number: 2018X079) and adhered to the Animals in Research: Reporting In Vivo Experiments (ARRIVE) guidelines.

### Cell culture

Patient-derived CRC cells (CRC101, CRC102, and CRC108) were obtained and maintained as previously described [7]. All cells were authenticated with STR profiling at Genetic Testing Center of Shanghai (Shanghai, China), and they resembled the genetic and biological characteristics of the original tumors. Isogenic cell lines of DKs-5 and DKO-3 were derived from DLD1 colon cancer cells and HK2-10 and HKe-3 from HCT116 cells, in which wt*Kras* or mu*Kras* alleles have been disrupted by targeted homologous recombination [15]. All cells were tested for mycoplasma before magnetic-activated cell sorting (MACS) and during follow-up studies using a MycoAlert mycoplasma detection kit (Lonza #LT07-118; Basel, Switzerland). MACS was used to sort CDCP1+ cells.

### Xenograft studies

Subcutaneous xenograft tumor models were established based on 4-week–old female BALB/c-nu mice, while orthotopic tumor transplantation was performed in 4-week–old female NOG mice as previously described [7]. To generate orthotopic tumors, we injected suspensions of the indicated cells into the cecal wall after laparotomy. Transduction with a GFP-labeled lentivirus (Invitrogen, Carlsbad, CA, USA) facilitated monitoring of tumor growth, regression, and recurrence by intravital imaging. Tumor volume was measure as normalized photon flux. When tumor reached approximately 250 units, mice were randomized into the indicated treatment groups, and the mean tumor volumes of each group were similar before the treatment started. No mice were excluded after the treatment started. To assess distant recurrence, lungs were removed from mice with recurrent tumors, and GFP-positive metastases were visualized using an Olympus MVX10 dissecting microscope (Olympus, Tokyo, Japan). For subcutaneous xenografts, the indicated cells suspended in 100 μl Hanks’ buffered saline solution were injected subcutaneously in the axillary region of the right chest in BALB/c-nu/nu mice. Tumor volume was measured every 2 days, starting from day 4 postinjection, to monitor the extent of tumor growth, regression, and recurrence. Tumor volume was calculated using the formula 1/2(Length × Width^2^). When tumor reached approximately 2 cm in diameter, mice were randomly divided into the indicated groups, and the mean tumor volumes of each group were similar before the treatment started. No mice were excluded after the treatment started. To determine the effects of FOLFOX, tumor-bearing mice were treated with IP oxaliplatin 6 mg/kg, followed 2 h later by 5-FU 50 mg/kg and folinic acid 90 mg/kg (all obtained from Sigma-Aldrich, Dorset, UK), twice weekly for 2 weeks. To chronically label tumors with EdU, mice were administrated with 0.82 mg/ml EdU (Invitrogen) in drinking water protected from light. For CidU/IdU labeling, CidU or IdU (Sigma-Aldrich) was dissolved in sterile 0.9% saline to prepare a 15 mg/ml solution, and mice were injected intraperitoneally with CidU (100 mg/kg/day) in 1 day with 2 h intervals and with IdU (300 mg/kg/day) 2 h before being killed.

### Transgenic mice

*B6;129S6-Gt(ROSA)26Sort*^*m9(CAG-tdTomato)Hze/J*^*(Rosa-CAG-LSL-tdTomato)* mice were purchased from the Jackson Laboratory (Bar Harbor, ME, USA). *pTRE-Kras*^*G12D*^, *CDCP1-Cre*^*ERT2*^, and *Fabpl*^*-4X@132*^*-rtTA-3×(IRES-Apc*.*3374)* mice were purchased from Cyagen Biosciences Company (Santa Clara, CA, USA). For the *pTRE-Kras*^*G12D*^ transgene, a fragment containing mutant murine *Kras*^*G12D*^ cDNA was directly inserted into the EcoRI/NheI site of pTRE-Tight. For the *Fabpl*^*-4X@132*^*-rtTA-3×(IRES-Apc*.*3374)* transgene, the murine *Fabpl*^*-4X@132*^ cDNA (nucleotides 2,596 to 121 of the rat *Fabpl* gene, with four additional tandem repeats of its nucleotides 2,172 to 2,133 added at nucleotide 2,132) and the *rtTA* open reading frame excised from *pUHG15-1-rtTA* (a gift from Dr. Michael Rosenberg, Glaxo Wellcome) were cloned into a modified pUC19 plasmid using the restriction sites within the oligos, after which three *IRES-Apc*.*3374* repeats (*Apc*.*3374*, an shRNA oligonucleotide targeting the *Apc* sequence: 5′-CTCGAGAAGGTATATTGCTGTTGACAGTGAGCGCCCCAATAAATTACAGTCTTATAGTGAAGCCACAGATGTATAAGACTGTAATTTATTGGTGTTGCCTACTGCCTCGGAATTC-3′) was placed downstream of the *Fabpl*^*-4X@132*^*-rtTA* cassette so that expression of *rtTA* and *Apc*.*3374* was directed to colonic epithelia. For the *CDCP1-Cre*^*ERT2*^ transgene, a 529-bp promoter fragment corresponding to the murine *CDCP1* genomic sequence 553/25 relative to the start codon was amplified from 129/SvEv genomic DNA. Amplicons were cloned into a modified pUC19 plasmid using the restriction sites within the oligos, after which *Cre*^*ERT2*^ cDNA, followed by bovine growth factor polyadenylation sequence, was placed downstream of the CDCP1 promoter fragment. *pTRE-Kras*^*G12D*^, *CDCP1-Cre*^*ERT2*^, and *Fabpl*^*-4X@132*^*-rtTA-3×(IRES-Apc*.*3374)* mice were produced by injecting the constructs into FVB/N blastocysts according to a standard protocol. Transgenic founders were screened by PCR analysis of genomic DNA. FCT mice (three weeks old, female) were bred and maintained in a pathogen-free facility. Dox was administered in drinking water (2 g/l in 20 g/l sucrose) to induce the formation of CRCs within 14 weeks. Tumor behavior from week to week was assessed as growth, regression, or relapse. The tumor’s developmental stage was monitored over time by colonoscopy every week following drug treatment or withdrawal. *Cre* recombination for lineage tracing was achieved by intraperitoneal injection of 5 mg tamoxifen (Sigma-Aldrich; dissolved in sesame oil).

### Optical colonoscopy

Mice were anesthetized using isoflurane and immobilized on a surgical platform. The colon was flushed with warm PBS. A small 1.5-mm rigid endoscope in an operating sheath was inserted through the anus into the colon. The colon was insufflated with air using a small pump, and photographs were obtained as the endoscope was withdrawn. This procedure took about 5 minutes on average. The colon reached maximal distention during each procedure because a small reduction in air flow resulted in partial collapse; consequently, the cross-sectional area was relatively constant overtime. Tumor behavior from week to week was assessed as growth, regression, or relapse. The tumor’s developmental stage was monitored over time by colonoscopy every week following drug treatment or withdrawal.

### Intravital imaging

Phoenix ecotropic packaging cells were transfected with *pBABE-GFP* using Effectene (QIAGEN, Hilden, Germany) according to the manufacturer’s instructions. 24 h later, cells were washed twice, and 1.5 ml of DMEM plus 10% FBS was added per well. Virus was collected after 48 h and centrifuged to remove any cells, and supernatant was stored at –80°C. Primary tumor cells were infected with 1 ml of virus and 4 μl polybrene (4 mg/ml). The next day, the media were replaced, and cells were subsequently selected with puromycin. After the transfected cells were orthotopically implanted into NOG mice, tumor growth was monitored via intravital imaging at the indicated time points. For intravital imaging, the mice were anesthetized with 2% isoflurane and placed in the IVIS-50 chamber (Caliper Life Sciences, Hopkinton, MA, USA) in the supine position, where they were maintained with isoflurane administered through nose-cone ports inside the chamber. The chamber temperature was kept constant at 37°C throughout the procedure. Light emission was collected with filters for green fluorescence and background fluorescence, and images were processed using Living Image software (Caliper Life Sciences). Specific signal was reported as the ratio of the fluorescence photon flux in the region of interest/the fluorescence signal in a background region containing no cells or tumors (normalized photon flux). A signal was defined as positive when it was greater than the sum of the mean background signal plus 2 SDs of the background signal.

### Antibodies

The primary antibodies used for immunohistochemistry, immunofluorescence, immunoprecipitation, and western blotting analysis were as follows: CDCP1 (Cell Signaling Technology #4115; Danvers, MA, USA), CDCP1 (Abcam #188818; Cambridge, UK), IdU (Abcam #181664), BrdU (Abcam #6326), HA (Abcam #18181), myc (Abcam #32072), Kras (Santa Cruz Biotechnology #sc-30; Dallas, TX, USA), Flag (Sigma-Aldrich #F1084), malonyl-lysine (PTM Biolabs 901; Chicago, IL, USA), succinyl-lysine (PTM Biolabs 401), glutaryl-lysine (PTM Biolabs 1151), acetyl-lysine (PTM Biolab 104), Sirt5 (Cell Signaling Technology 8782), tubulin (Abcam 7291), and TPI (Abcam #96696). The secondary antibodies used for immunofluorescence were Alexa Fluor 488 or 594 goat anti-mouse (#A-11001, #A-11005) or rabbit anti-mouse (#A-11034, R37117) IgG from Thermo Fisher Scientific (Waltham, MA, USA). The HRP-conjugated secondary antibodies (#7076, #7074, and #7077) used for western blot were from Cell Signaling Technologies.

### Evaluation of immunohistochemical staining for CDCP1

The neoplastic areas of tumor specimens were examined for CDCP1 staining by two independent pathologists. Only the membrane-specific immunostain in tumor parenchyma cells was considered as positive for CDCP1. CDCP1 staining intensity were scored as follows: negative, no staining; low, positive staining present in 2% to 10% of the total cells; high, positive staining in more than 10% of the total cells.

### MACS

Tumor cell suspension was rinsed with PBS after detaching and centrifuged at 1,100 rpm for 10 min. Pelleted cells were resuspended in PBS with a 1:100 dilution of the anti-CDCP1 monoclonal antibody (TA502228; Origene, Rockville, MD, USA). The cell–antibody complex was incubated at room temperature for 30 min and rinsed twice with PBS. This step was followed by 15 min incubation at 6°C–12°C with a 1:5 dilution of goat anti-mouse IgG microbeads (Miltenyl Biotec GmbH, Bergisch Gladbach, Germany). After the mixture was subjected to a magnetic field, CDCP1+ cells adhered to IgG microbeads and the remaining, unadhered CDCP1– cells were separated using a MiniMACS separation system (Miltenyl Biotec GmbH). Finally, we took samples and detected the purity of CDCP1+ and CDCP1– cells with flow cytometry. Isolated CDCP1+ cells were grown to form tumor spheres in DMEM/F12 medium containing 20 ng/ml EGF (Sigma-Aldrich), 10 ng/ml FGF2 (Sigma-Aldrich), B27 (Gibco, Gaithersburg, MD, USA), and ITSS (Roche, Basel, Switzerland).

### Immunoprecipitation

Cells were collected and lysed in lysis buffer supplemented with protease inhibitors, incubated on ice for 15 min, and cleared by centrifugation at 8,000 × *g* at 4°C for 15 min. Total protein lysates were subjected to immunoprecipitation with the agarose-immobilized antibody (0.01 mg of anti-Sirt5, TPI, Flag, HA, myc, or isotype control antibody) for overnight at 4°C.

### OFFGel prefractionation of TPI immunoprecipitates

Isoelectric point-based OFFGel prefractionation for immunoprecipitates was carried out in 3 technical replicates as specified by the manufacturer. Briefly, 2 mg of TPI immunoprecipitates was used for OFFGel prefractionation using 3100 OFFGel fractionator (Agilent Technologies, Tokyo, Japan). Proteins were separated using IPG strips (pH 3–10, 13 cm, Agilent Technologies) focused for 20 kV h with a maximum current of 50 μA and maximum voltage set to 4,500 V. Samples were shifted to hold step at a specific voltage (500 V), and a current of 20 μA was sustained for 40 h. During the focusing, oil was added to the electrodes to prevent evaporation. After successful fractionation, 12 liquid fractions were recovered and pooled prior to in-solution digestion.

### In-solution trypsin digestion

Recovered fractionated proteins were precipitated with acetone and assayed using the BCA method (Pierce, Rockford, IL, USA). Acetone-precipitated proteins (approximately 50 μg) were denatured in 8 M urea, 500 mM Tris-HCl (pH 8.5) with protease and phosphatase inhibitor cocktails (Roche, Mannheim, Germany), followed by reduction with 5 mM tris(2-carboxyethyl)phosphine (TCEP) for 30 min to confirm fully reduced protein sample before digestion. Cysteine residues were alkylated with 10 mM iodoacetamide for 20 min in the dark. To maintain trypsin activity, samples were diluted to a final concentration of 2 M urea in 100 mM Tris-HCl (pH 8.5), prior to digestion with trypsin. For endopeptidase digestion, modified trypsin (Promega, Madison, WI, USA) was added at 50:1 (protein/protease mass ratio) along with 1 mM CaCl_2_ and incubated overnight in a thermo-shaker at 600 rpm at 37°C. Digested peptide solution was acidified using 90% formic acid to a final pH of 3 and enriched using a stage tip.

### LC-MS/MS

Digested peptide samples were desalted with a C8 OptiPak column (Optimize Technologies) and analyzed by liquid chromatography-electrospray tandem mass spectrometry (LC-ESI/MS/MS) on a Thermo LTQ Orbitrap Hybrid FT mass spectrometer. Positive-ion mass spectra were acquired in the reflectron mode. Ions selected for MS/MS were subsequently placed on an exclusion list using an isolation width of 1.6 Da, a low-mass exclusion of 0.8 Da, and a high-mass exclusion of 0.8 Da. Tandem mass spectra were extracted by Readw.exe version 3.0. All MS/MS samples were analyzed using Mascot (Matrix Science, London, UK) data explorer software, and Scaffold (version Scaffold_2.1.03; Proteome Software, Inc., Portland, OR, USA) was used to validate MS/MS-based peptide and protein identifications.

### Intracellular ROS, NADPH level, and GSH/GSSG ratio measurements

Total intracellular ROS was determined by staining the cells with carboxy-H2DCFDA (Invitrogen); 2 × 10^5^ cells were incubated with 10 mM carboxy-H2DCFDA for 30 min at 37°C. Cells were washed and analyzed by flow cytometry (BD FACSCanto; BD Biosciences, San Jose, CA, USA). Intracellular NADPH level and GSH/GSSG ratio were determined using fluorimetric SensoLyte NADP/NADPH Assay (AnaSpec, Fremont, CA, USA) and GSH/GSSG-Glo (Promega) as per manufacturer’s recommendations, respectively.

### Ras activity measurement

Measurement of Ras activity was performed using the Active Ras Detection Kit (#8821, Cell Signaling Technology) as per manufacturer’s recommendations.

### RCB analysis

To quantify tumor response, RCB was assessed at the time of surgery, which takes into account parameters such as tumor bed area, overall cellularity, and lymph node involvement. We separated tumors into three groups based on RCB class. The first group contained RCB 0 and I tumors, which have the lowest RCB. The second group contained RCB II tumors. The third group contained RCB III tumors, which exhibited minimal response or progression in the colon and/or lymph nodes at the time of surgery and have the highest burden. Because RCB 0 and I tumors are associated with a low risk of recurrence, these classes were combined for our analysis.

### Cell apoptosis assays

Cell apoptosis was determined by flow cytometry after double staining with Annexin-V and PI (BD Biosciences). All assays were repeated at least three times.

### MS for metabolic intermediates

For metabolite collection, the media of the treated cells was aspirated, and the cells were washed twice with 75 mM ammonium carbonate (pH 7.4). Afterwards, the cellular metabolism got quenched by immersion into liquid nitrogen. Subsequently, intracellular metabolites were extracted twice with 70% ethanol at 75°C for 3 min. Cell debris in the metabolite extracts was removed by centrifugation at 13,000 rpm for 1 min. Metabolite extracts were dried in a speedvac at 0.12 mbar pressure at room temperature until the samples were completely dry (4–6 h). For mass spectrometric analysis, dried metabolite samples were resuspended in 80 μl H_2_O. Quantitative targeted analysis of selected metabolite levels and analysis of mass isotopomer distributions were performed using ultra high-pressure chromatography-coupled MS/MS as described before [40]. Abundance of mass isotopomers for all metabolites was corrected for natural abundance of ^13^C as previously described [41].

### Analysis of the PPP activity

PPP activity was determined by following the procedure as described [42]. Briefly, 10^6^ cells were grown in a 6-cm culture plate in sodium-bicarbonate–free RPMI medium supplemented with 10% FBS, 20 mM HEPES, 5 mM glucose, and 0.2 μCi of [1-^14^C]-glucose or [6-^14^C]-glucose (American Radiolabeled Chemicals, St. Louis, MO, USA). The cells were placed in a closed glass vial, the center of which was covered with filter paper soaked in 100 μl of 5% KOH, and then incubated at 37°C for 4 h. The filter paper was removed, and the radioactivity was determined using an LS 6500 Multi-Purpose Scintillation Counter (Beckman Coulter, Brea, CA). PPP activity was calculated as the difference between the radioactivity levels of samples obtained from [1-^14^C]-glucose and samples obtained from [6-^14^C]-glucose, normalized to cell number.

### Metabolic analysis of PPP flux

The indicated cells were then incubated for 3 h with fresh medium supplemented with 5 mM glucose and 2 mM glutamine before replacing the unlabeled medium with the corresponding labeled medium (supplemented with 5 mM [1,2-^13^C]-glucose and 2 mM glutamine). After incubation for 4 h, the medium was aspirated, and the cells were washed twice with cold PBS. Prechilled 80% aqueous methanol (–80°C; 1 ml) was quickly added to each well. Cells were scraped off the well and transferred into microcentrifuge tubes. The extraction was repeated, and both fractions were combined and centrifuged at 20,000 × *g* for 2 min. Supernatants were collected and dried by speedvac. Cell extracts obtained were analyzed for relative abundance of ^13^C-metabolites by LC-MS using scheduled selective reaction monitoring (SRM) for each metabolite of interest, with the detector set to negative mode. Prior to injection, dried extracts were reconstituted in LC-MS–grade water. LC separation was achieved by reverse-phase ion-pairing chromatography as described [40]. Extracted metabolite concentrations were calculated from standard metabolite build-up curves using natural ^12^C synthetic metabolites and normalized against cell number, as well as the internal ^13^C-labeled metabolite standards added at the time of metabolite extraction. Calculations for relative percentage of PPP flux were as described [43].

### TPI enzyme activity assay

Activity of TPI in cell-free protein extracts was determined in an enzyme-coupled reaction with glycerol 3-phosphate dehydrogenase. Optical density measurements at 340 nm were used to detect NADH to NAD+ oxidation upon adding the TPI substrate GAP and recorded in 12-s intervals in a spectrophotometer (Amersham Pharmacia Biotech, Piscataway, NJ, USA). Km and Ki values were determined by generating saturation curves with G3P and PEP, respectively.

### Enzymatic assays of GAPDH, PGM, PGK, PK, and enolase

GAPDH enzymatic activity was estimated with KDalert GAPDH Assay (Ambion, Austin, TX, USA). Increase in fluorescence was measured by use of a SpectraMax M2 spectrofluorometer (Molecular Devices, Sunnyvale, CA, USA) with excitation at 560 nm and emission at 590 nm and quantified against the calibration curve. PGK activity was determined by enzymatic assay, spectrophotometrically following the oxidation of β-NADH at an optical density of 340 nm. We measured PGK activity using the coupled reaction with GAPDH from the previous step of the glycolytic pathway, as described in detail earlier. NADH consumption was monitored as a function of time by following the absorbance at 340 nm. The rate of NADH consumption was measured at various concentrations of the substrate 3-phosphoglycerate and plotted as a function of substrate concentration to extract the enzyme activity. PGM activity was assayed using a previously described protocol [44]. Briefly, samples were sonicated and cleared by centrifugation (13,500 rpm, 4°C). Then, 20 mg total protein was mixed with assay buffer (50 mM K_2_H_2_PO_4_ [pH 7.5], 10 mM MgCl_2_, 5 mM EDTA [pH 8]) containing 0.5 mM NADP+, 1.5 units/ml glucose-6-phosphate dehydrogenase, 5 mM glucose-1-phosphate, and 0.02 mM glucose 1,6-bisphosphate to a total volume of 150 μl. Reagents were from Sigma-Aldrich. Absorbance at 340 nm was measured kinetically to assess the amount of NADPH produced using a SpectraMax i3 (Molecular Devices). Enolase activity was assayed spectrophotometrically at 240 nm at room temperature as an increase in PEP concentration. The reaction was measured in 50 mM imidazole–HCl buffer (pH 6.8), containing 3 mM MgSO_4_, 0.4 M KCl, and 1 mM 2-phosphoglycerate acid (PGA) substrate. One unit of enolase activity is defined as the amount of protein which catalyses the synthesis of 1 μM PEP min^–1^ under these conditions. The molar absorption coefficient was taken as 1,520 cm^2^ mol^–1^. PK activity assay was performed using a PK activity assay kit (BioVision, Milpitas, CA, USA) according to the manufacturer's protocol. Cell extracts were prepared by lysing cells with 4 volumes of pyruvate assay buffer and spinning at 15,000 rpm for 15 min at 4°C to remove insoluble material. Cell extracts were added into a 96-well flat bottom plate. The volume was adjusted to 50 μl/well with pyruvate assay buffer. Then, 50 μl of reaction mixture (46 μl of pyruvate assay buffer, 2 μl of pyruvate probe, and 2 μl of enzyme mixture) per well were added and mixed well. The absorbance A_570_ nm was scanned once per minute for 40 min at room temperature. At the same time, a standard curve of nmol/well versus A_570_ nm readings was plotted. Then, the sample readings were applied to the standard curve to obtain the amount of pyruvate in the sample wells. The rate of pyruvate yield was normalized by the amount of total proteins in the lysate or the amount of PK.

### Cell-cycle analysis

FACS-sorted CDCP1+ CSCs were incubated for 45 min at 37°C with 20 μg/ml Hoechst 33342 (Invitrogen) in DMEM/F12 medium containing 20 ng/ml EGF (Sigma-Aldrich), 10 ng/ml FGF2 (Sigma-Aldrich), B27 (Gibco), and ITSS (Roche). Pyronin Y (Sigma-Aldrich) was then added at 1 μg/ml, and the cells were incubated for another 15 min at 37°C, washed, and immediately analyzed using a MoFlow cytometer (Cytomation, Fort Collins, CO, USA) and FlowJo program (Cytomation).

### Flow cytometry

For cell-surface–marker analysis, the indicated cells were incubated with the primary anti-CDCP1-PE antibodies (#324006; Biolegend, San Diego, CA, USA) and secondary fluorescent-conjugated antibodies. All cells were analyzed on a FACSCalibur flow cytometer (BD Biosciences). Fluorescent intensities were point plotted on 2-axis graphs or histograms using Cell-Quest software (Becton Dickinson, Franklin Lakes, NJ, USA). All fluorescence-activated cell-sorter analyses were paired with matched isotype control. Dead cells were excluded with PI (1:1,000).

### Statistical analysis

Statistical analyses were performed with SPSS software (SPSS, Chicago, IL, USA). Survival plot *P*-value was generated using a log-rank test. The effect of CDCP1 status at the time of primary surgical resection was assessed by multivariable survival analysis according to the Cox proportional hazards regression model, adjusting for potential confounding prognostic factors. For all other comparisons, a two-tailed unpaired *t* test was used. *P* < 0.05 was considered to indicate statistical significance throughout the study. The investigators were not blinded to allocation during experiments and outcome assessment except for immunohistochemical analyses. For each data set, the data meet the assumptions of the statistical test used, as determined by distribution and variance. No statistical method was used to predetermine sample size. The sample size for all experiments (in vitro and in vivo) was not chosen with consideration of adequate power to detect a prespecified effect size. For in vitro studies, all experiments were independently repeated three times in triplicate. All completed experiments are reported. No samples were fully processed for metabolomic, western blot, or immunohistochemical analysis and then excluded. For in vivo experiments, efforts were made to achieve the scientific goals of this study with the minimum number of animals. With respect to randomization, for animal experiments, tumor-bearing mice of similar tumor burden were equally divided into the control and experimental groups for subsequent drug treatment. No experimental samples were excluded throughout this study, with the exception that animals that experienced unexpected, acute illness and/or injury were removed per the veterinarian's order.

## Supporting information

S1 Fig CDCP1+ CSCs in mu*Kras* CRCs survive chemotherapy. (A) DKs5, DKO3, HK2-10, and HKe3 cells were persistently treated with oxaliplatin (1 μM) and analyzed for CDCP1 expression by flow cytometry at the indicated time points. Dead cells were detected by 7AAD staining. (B) DKO3 and HKe3 cells were persistently treated with cetuximab (5 μM) and analyzed for CDCP1 expression by flow cytometry at the indicated time points. Dead cells were detected by 7AAD staining. (C) BALB/c-nu mice received FOLFOX treatment twice weekly for 2 weeks when CRC108 and CRC102 epidermal xenografts reached approximately 2 cm in diameter (left panels); macroscopic examination revealed regression within 2 weeks (middle panels) and subsequent relapse within 4 weeks (right panels). (D) CRC108 cells were treated with 5-FU (5 μM), after which CDCP1 mRNA expression was quantified by qPCR at the indicated time points. (E) Representative examples of CDCP1 scoring of primary tumors from patients with mu*Kras* CRCs: CDCP1^negative^, CDCP1^low^, and CDCP1^high^. (F) Kaplan–Meier graph showing the fraction of patients with liver-recurrence–free survival for the patients with mu*Kras* CRCs, dichotomized by CDCP1 expression status of primary tumors. (G) CDCP1 expression in primary wt*Kras* CRCs prior to treatment versus RCB. *P*-value was determined by the log-rank test. Sample size is indicated in parentheses. Values shown are mean ± SD. *P*-values were calculated by two-tailed *t* test unless otherwise indicated. **p < 0.05. Bar: 50 μm. Underlying data are available in S1 Data. CDCP1, CUB-domain–containing protein 1; CRC, colorectal carcinoma; CSC, cancer stem cell; FOLFOX, folinic acid + fluorouracil + oxaliplatin; *Kras*, Kirsten rat sarcoma viral oncogene homolog; mu*Kras*, mutant *Kras*; qPCR, quantitative PCR; RCB, residual cancer burden; wt*Kras*, wild-type *Kras*; 5-FU, 5-fluoropyrimidine; 7AAD, 7-aminoactinomycin.
(TIF)

S2 Fig CDCP1+ CSCs represent the driving force behind recurrence in mu*Kras* CRCs. (A) FCT mice were fed with Dox-containing water for 2 weeks starting at 3 weeks of age. Total RNA was prepared from the indicated tissues, and *Kras*^*G12D*^ expression was measured by qPCR with transgene-specific primers. (B–C) Total colonic RNA and colon tissue lysates were prepared from FCT mice fed with Dox-containing water for the indicated times. *Kras*^*G12D*^ expression and colonic Ras activity were measured by qPCR (B; *n* = 3 per time point) and Raf-RBD pull-down assays (C; *n* = 3 per time point). (D) Colonic Apc expression in control *Fapbl*^*4X@-132*^*-rtTA* and *Fapbl*^*4X@-132*^*-rtTA-3×(IRES-Apc*.*3374)* mice. The experiments in A–D were independently repeated three times in triplicate. (E) Colonoscopic examination (upper panels) and HE staining (bottom panels) of FCT tumors after FOLFOX administration or withdrawal at the indicated time points. (F) Representative immunofluorescent images (left panel) and quantification (right panel) of CDCP1+tdTomato+ cells 24 h post-tamoxifen injection in FCT mice (*n* = 3). Arrowheads indicate the CDCP1+tdTomato+ cells. (G) Representative images (left panels) and quantification (right panel) of tdTomato labeling in FCT primary tumors (*n* = 3) versus relapsed tumors after FOLFOX withdrawal (*n* = 3). Values shown are mean ± SD. *P*-values were calculated by two-tailed *t* test unless otherwise indicated. **p < 0.05. Bar: 50 μm. Underlying data are available in S1 Data. Apc, adenomatous polyposis coli; CDCP1, CUB-domain–containing protein 1; CRC, colorectal carcinoma; CSC, cancer stem cell; Dox, doxycycline; *Fapbl*, liver-type fatty-acid–binding protein; FCT, *Fabpl*^*4X@132*^*-rtTA-3×(IRES-Apc*.*3374)*:*tet-Kras*^*G12D*^:*CDCP1-creERT2*:*Rosa26 CAG-loxP-stop-loxP-tdTomato*; FOLFOX, folinic acid + fluorouracil + oxaliplatin; HE, hematoxylin–eosin; *IRES*, internal ribosome entry site; *Kras*, Kirsten rat sarcoma viral oncogene homolog; mu*Kras*, mutant *Kras*; qPCR, quantitative PCR; Raf, murine leukemia viral oncogene homolog; Ras, rat sarcoma viral oncogene homolog; RBD, Ras-binding domain; *rtTA*, reverse tetracycline-controlled transactivator; td, tandem dimer
(TIF)

S3 Fig Oxidative PPP contributes to NADPH production in CDCP1+ CSCs from mu*Kras* CRCs. (A) The ratios of [NADPH/NAPD+] in CRC108-derived CDCP1+ cells transfected with control shRNA or shRNA targeting G6PD, 6PGD, ME1, MTHFD1, MTHFD2, IDH1, or IDH2, respectively. (B) Percentage of central carbon flux from glucose to lactate flowing through the PPP in CDCP1+ and CDCP1– fractions isolated from DKs5 and HK2-10 cells. Flux was determined from the relative enrichment of doubly versus singly [^13^C]-labeled lactate, pyruvate, and 3-phosphoglycerate, as measured using negative mode LC-MS of extracts from cells fed with [1,2-^13^C]-glucose. (C) ROS levels and the ratios of [NADPH/NAPD+] in DKs5- or HK2-10–derived CDCP1+ cells with G6PD or TK/TA KD. All experiments were independently repeated three times in triplicate. Values shown are mean ± SD. A two-tailed unpaired *t* test was used to compare experimental groups. **p < 0.05. Underlying data are available in S1 Data. CDCP1, CUB-domain–containing protein 1; CRC, colorectal carcinoma; CSC, cancer stem cell; G6PD, glucose-6-phosphate dehydrogenase; IDH, isocitrate dehydrogenase; KD, knockdown; *Kras*, Kirsten rat sarcoma viral oncogene homolog; LC-MS, liquid chromatography-triple quadrupole mass spectrometry; ME1, malic enzyme 1; MTHFD, methylenetetrahydrofolate dehydrogenase; mu*Kras*, mutant *Kras*; PPP, pentose phosphate pathway; ROS, reactive oxygen species; shRNA, short hairpin RNA; TA, transaldolase; TK, transketolase; 6PGD, 6-phosphogluconate dehydrogenase.
(TIF)

S4 Fig Sirt5 demalonylates TPI to repress its enzyme activity. (A) The enzyme activities of TPI, GAPDH, PGK, PGM, enolase, and PK were compared between CDCP1– and CDCP1+ fractions from DKs5, HK2-10, and CRC102 cells. The experiments were independently repeated three times in triplicate. (B) The expression level of endogenous TPI was compared between CDCP1– and CDCP1+ fractions from DKs5, HK2-10, and CRC102 cells. (C) TPI malonylation was compared between CDCP1– and CDCP1+ fractions from wt*Kras* and mu*Kras* CRC cells. (D) The HA-H158Y mutant was transfected into DKs5- or HK2-10–derived CDCP1+ cells. The association of endogenous TPI with HA-H158Y was determined by co-IP. (E) Annotation of a representative tandem mass spectrum of trypsin-digested TPI showing malonylation of K56, K122, and K231 upon Sirt5 KD in CRC108-derived CDCP1+ cells. (F) The indicated HA or Flag-tagged TPI proteins were overexpressed in DKs5-derived CDCP1+ cells with stable TPI KD. The presence of ectopically expressed and endogenous proteins was verified by western blot. Values shown are mean ± SD. A two-tailed unpaired *t* test was used to compare experimental groups. Underlying data are available in S1 Data. CDCP1, CUB-domain–containing protein 1; CRC, colorectal carcinoma; GAPDH, glyceraldehyde -3-phosphate dehydrogenase; HA, hemagglutinin; IP, immunoprecipitation; KD, knockdown; *Kras*, Kirsten rat sarcoma viral oncogene homolog; mu*Kras*, mutant *Kras*; PGK, phosphoglycerate kinase; PGM, phosphoglucomutase; PK, pyruvate kinase; Sirt5, silent mating type information regulation 2 homolog 5; TPI, triosephosphate isomerase; wt*Kras*, wild-type *Kras*.
(TIF)

S5 Fig Activation of oxidative PPP in CDCP1+ CSCs contributes to recurrence in mu*Kras* CRCs. (A) Schematic representation of the parental (upper), intermediate (middle), and final dual-promoter (lower) lentiviral vectors. (B–C) CRC108 cells labeled with GFP were transfected with the dual-promoter lentiviral vectors in S5A Fig. HA-TPI and TPI expression in CDCP1– and CDCP1+ subpopulations were analyzed at 24 h after Dox treatment (1 μg/ml) (B); the frequency of CDCP1+ cells was analyzed at 72 h after Dox treatment (1 μg/ml) (C). The experiments were independently repeated three times in triplicate. (D) Upon visible tumor formation after orthotopic implantation into 4-week–old female NOG mice, animals were randomized to receive FOLFOX (twice weekly for 2 weeks) in the presence (*n* = 35) or absence (*n* = 33) of Dox. Tumor growth and regression were monitored after implantation (*n* = 3 per time point). Values shown are mean ± SD. A two-tailed unpaired *t* test was used to compare experimental groups. **p < 0.05. Underlying data are available in S1 Data. CDCP1, CUB-domain–containing protein 1; CRC, colorectal carcinoma; CSC, cancer stem cell; Dox, doxycycline; FOLFOX, folinic acid + fluorouracil + oxaliplatin; GFP, green fluorescent protein; HA, hemagglutinin; *Kras*, Kirsten rat sarcoma viral oncogene homolog; mu*Kras*, mutant *Kras*; NOG, nonobese diabetic (NOD)/Shi-scid Il2rg^null^; PPP, pentose phosphate pathway; TPI, triosephosphate isomerase.
(TIF)

S6 Fig Characterization of the specificity of the α-malTPI(K56) antibody. (A) Specificity of antibody against malonylated K56 of TPI was determined by dot blot assay. Nitrocellulose membrane was spotted with different amounts of malonyl-K56 peptide or unmodified peptide and detected with a site-specific antibody against K56 malonylation [α-malTPI(K56)]. (B) Immunoprecipitated Flag-tagged WT TPI or its K56A/K56E mutants were detected by the α-malTPI(K56) antibody. (C) Malonylated K56 peptide, but not the unmodified peptide, competed with malonylated TPI. malTPI, malonylated TPI; TPI, triosephosphate isomerase; WT, wild type.
(TIF)

S1 Table Multivariate HRs from Cox proportional hazards regression models of CDCP1 expression level and lung- or liver-relapse–free survival for mu*Kras* CRCs, adjusting for patient characteristics. CDCP1, CUB-domain–containing protein 1; CRC, colorectal carcinoma; *Kras*, Kirsten rat sarcoma viral oncogene homolog; mu*Kras*, mutant *Kras*.
(XLS)

S2 Table TPI-interacting proteins in CDCP1+ cells. The Flag-TPI vector was transfected into CRC102-derived CDCP1+ cells, after which the cells were lysed and precleared prior to IP. The IP was performed using a Flag rabbit monoclonal antibody chemically coupled to agarose beads. OFFGel prefractionation was used to reduce sample complexity. Fractionated proteins were subjected to in-solution digestion before LC-MS/MS analysis. CDCP1, CUB-domain–containing protein 1; CRC, colorectal carcinoma; IP, immunoprecipitation; LC-MS/MS, liquid chromatography-tandem mass spectrometry; TPI, triosephosphate isomerase.
(XLS)

S1 Data Data used to generate the figures. (XLSX)

## Abbreviations

acetyl-K: lysine acetylation
Akt: RAC-alpha serine/threonine-protein Kinase
AktCA: catalytically active mutant of Akt
ALDH1A1: aldehyde dehydrogenase 1 family member A1
Apc: adenomatous polyposis coli
ARRIVE: Animals in Research: Reporting In Vivo Experiments
CAG: cytomegalovirus: early enhancer/chicken beta actin
cCDPC1: cleaved CDCP1
CD: cluster of differentiation
CDCP1: CUB-domain–containing protein 1
CidU: 5-Chloro-2′-deoxyuridine
CRC: colorectal carcinoma
creERT2: Cre recombinase-estrogen receptor T2
CSC: cancer stem cell
CUB: complement C1r/C1s, Uegf, Bmp1
DHAP: dihydroxyacetone phosphate
Dox: doxycycline
EdU: 5-ethynyl-2′-deoxyuridine
EGFR: epidermal growth factor receptor
ESA: epithelial-specific antigen
E4P: erythrose 4-phosphate
*Fabpl*: liver-type fatty-acid–binding protein
FACS: fluorescence-activated cell sorting
fCDCP1: full-length CDCP1
FCT: *Fabpl*^*4X@132*^*-rtTA-3×(IRES-Apc*.*3374)*:*tet-Kras*^*G12D*^:*CDCP1-creERT2*:*Rosa26 CAG-loxP-stop-loxP-tdTomato*
FOLFOX: folinic acid + fluorouracil + oxaliplatin
F6P: fructose-6-phosphate
GAP: glyceraldehyde 3-phosphate
GAPDH: glyceraldehyde-3-phosphate dehydrogenase
GFP: green fluorescent protein
glu-K: lysine glutarylation
GSH: glutathione
GSSG: glutathione disulfide
G6PD: glucose-6-phosphate dehydrogenase
HA: hemagglutinin
HEK: human embryonic kidney
HER2: human epidermal growth factor receptor 2
HIF: hypoxia-inducible factor
IDH1/2: isocitrate dehydrogenase 1 and 2
IdU: 5-Iodo-2′-deoxyuridine
IgG: immunoglobulin G
IP: intraperitoneal
*IRES*: internal ribosome entry site
KD: knockdown
Ki67: marker of proliferation Ki-67
Kras: Kirsten rat sarcoma viral oncogene homolog
LC-ESI/MS/MS: liquid chromatography-electrospray tandem mass spectrometry
LC-MS: liquid chromatography-triple quadrupole mass spectrometry
*loxP*: locus of crossover in P1
mAb: monoclonal antibody
MACS: magnetic-activated cell sorting
malTPI: malonylated TPI
mal-K: lysine malonylation
MCM2: minichromosome maintenance complex component 2
MEK: mitogen-activated protein kinase
MEKCA: catalytically active mutant of MEK
ME1: malic enzyme 1
MS: mass spectrometry
MTHFD1/2: methylenetetrahydrofolate dehydrogenase 1 and 2
mu*Kras*: mutant *Kras*
NAC: N-acetylcysteine
NOG: nonobese diabetic (NOD)/Shi-scid Il2rg^null^
PCNA: proliferating cell nuclear antigen
PEP: phosphoenolpyruvate
PGA: 2-phosphoglycerate acid
PGI: phosophoglucoisomerase
PGK: phosphoglycerate kinase
PGM: phosphoglucomutase
PI3K: phosphoinositide 3-kinase
PK: pyruvate kinase
PKCδ: protein kinase C-delta
PPP: pentose phosphate pathway
PTM: posttranslational modification
RCB: residual cancer burden
RFS: recurrence-free survival
ROS: reactive oxygen species
*Rosa*: ROSAβgeo26
rtTA: reverse tetracycline-controlled transactivator
R5P: ribose 5-phosphate
shRNA: short hairpin RNA
Si-KRAS: KRAS siRNA
siRNA: small interfering RNA
Sirt5: silent mating type information regulation 2 homolog 5
SRC: proto-oncogene tyrosine-protein kinase SRC
SRM: selective reaction monitoring
succ-K: lysine succinylation
S7P: sedoheptulose 7-phosphate
TA: transaldolase
TCA cycle: the citric acid cycle
TCEP: tris(2-carboxyethyl)phosphine
td: tandem dimer
TK: transketolase
TPI: triosephosphate isomerase
WB: western blot
WT: wild type
wt*Kras*: wild-type *Kras*
xPG: 2/3-phosphoglycerate
1,3PG: 1,3-biphosphoglycerate
5PRA: 5-phosphoribosylamine
5-FU: 5-fluoropyrimidine
6PG: 6-phosphogluconate
6PGD: 6-phosphogluconate dehydrogenase
6PGL: 6-phosphoglucono-D-lactone
7AAD: 7-aminoactinomycin
