# Supplementary figures and images for: Targeting oxidative pentose phosphate pathway prevents recurrence in mutant Kras colorectal carcinomas
Source: PLoS Biol. 2019 Aug 28;17(8):e3000425. doi: 10.1371/journal.pbio.3000425 (PMC6736310; doi:10.1371/journal.pbio.3000425)

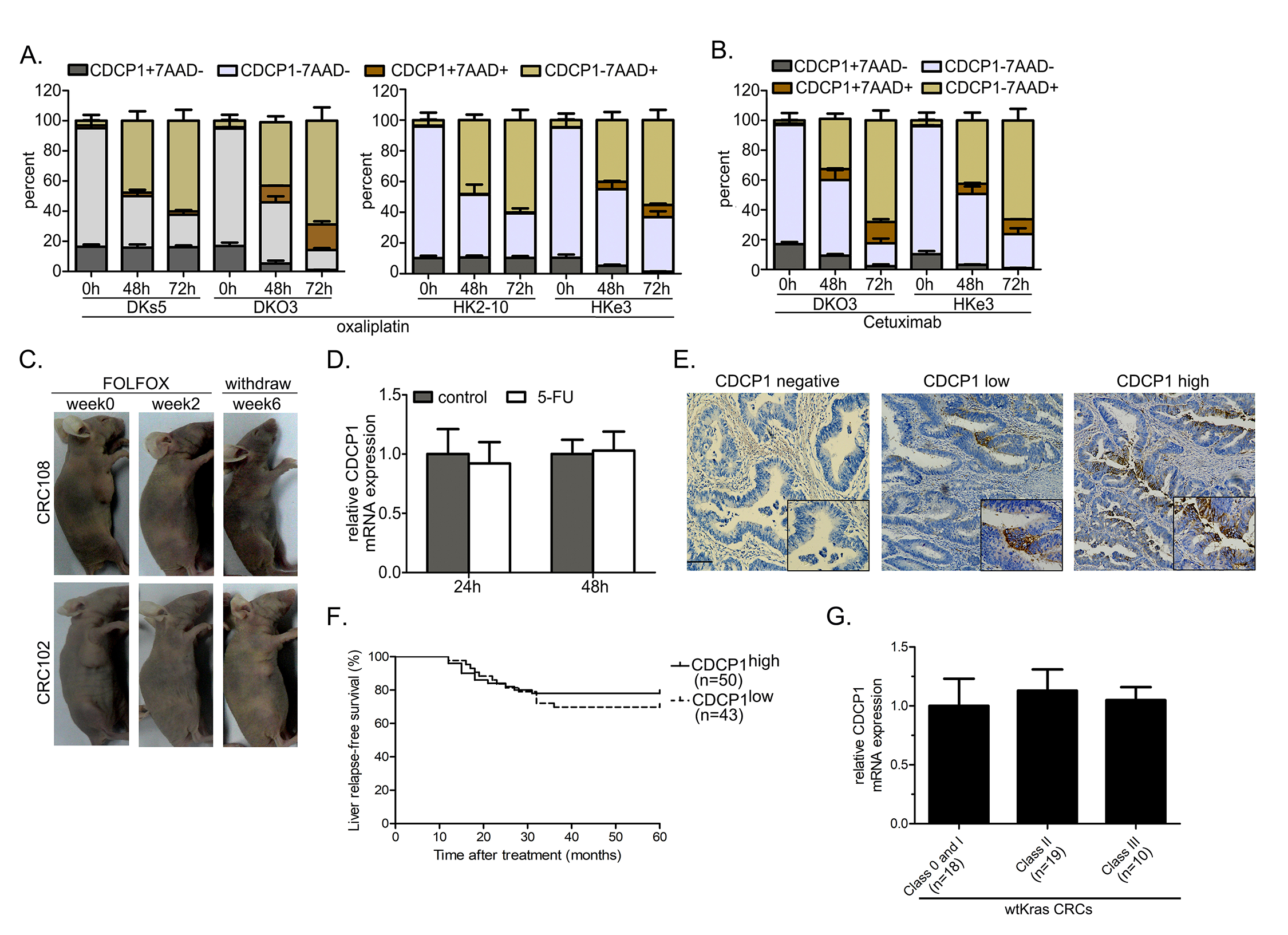

Supplement: S1 Fig — (A) DKs5, DKO3, HK2-10, and HKe3 cells were persistently treated with oxaliplatin (1 μM) and analyzed for CDCP1 expression by flow cytometry at the indicated time points. Dead cells were detected by 7AAD staining. (B) DKO3 and HKe3 cells were persistently treated with cetuximab (5 μM) and analyzed for CDCP1 expression by flow cytometry at the indicated time points. Dead cells were detected by 7AAD staining. (C) BALB/c-nu mice received FOLFOX treatment twice weekly for 2 weeks when CRC108 and CRC102 epidermal xenografts reached approximately 2 cm in diameter (left panels); macroscopic examination revealed regression within 2 weeks (middle panels) and subsequent relapse within 4 weeks (right panels). (D) CRC108 cells were treated with 5-FU (5 μM), after which CDCP1 mRNA expression was quantified by qPCR at the indicated time points. (E) Representative examples of CDCP1 scoring of primary tumors from patients with muKras CRCs: CDCP1negative, CDCP1low, and CDCP1high. (F) Kaplan–Meier graph showing the fraction of patients with liver-recurrence–free survival for the patients with muKras CRCs, dichotomized by CDCP1 expression status of primary tumors. (G) CDCP1 expression in primary wtKras CRCs prior to treatment versus RCB. P-value was determined by the log-rank test. Sample size is indicated in parentheses. Values shown are mean ± SD. P-values were calculated by two-tailed t test unless otherwise indicated. **p < 0.05. Bar: 50 μm. Underlying data are available in S1 Data. CDCP1, CUB-domain–containing protein 1; CRC, colorectal carcinoma; CSC, cancer stem cell; FOLFOX, folinic acid + fluorouracil + oxaliplatin; Kras, Kirsten rat sarcoma viral oncogene homolog; muKras, mutant Kras; qPCR, quantitative PCR; RCB, residual cancer burden; wtKras, wild-type Kras; 5-FU, 5-fluoropyrimidine; 7AAD, 7-aminoactinomycin. (TIF) [file pbio.3000425.s001.tif]

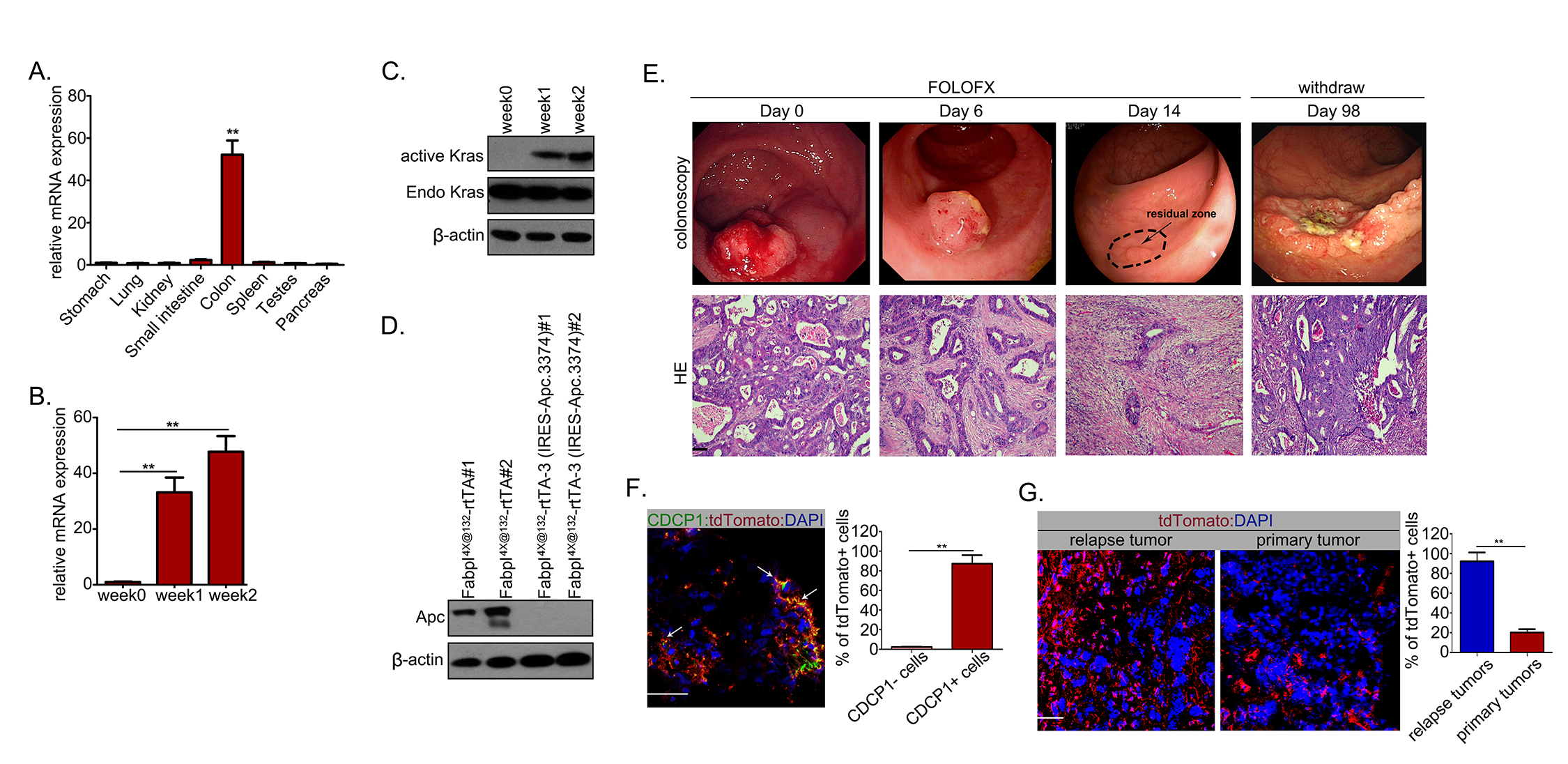

Supplement: S2 Fig — (A) FCT mice were fed with Dox-containing water for 2 weeks starting at 3 weeks of age. Total RNA was prepared from the indicated tissues, and KrasG12D expression was measured by qPCR with transgene-specific primers. (B–C) Total colonic RNA and colon tissue lysates were prepared from FCT mice fed with Dox-containing water for the indicated times. KrasG12D expression and colonic Ras activity were measured by qPCR (B; n = 3 per time point) and Raf-RBD pull-down assays (C; n = 3 per time point). (D) Colonic Apc expression in control Fapbl4X@-132-rtTA and Fapbl4X@-132-rtTA-3×(IRES-Apc.3374) mice. The experiments in A–D were independently repeated three times in triplicate. (E) Colonoscopic examination (upper panels) and HE staining (bottom panels) of FCT tumors after FOLFOX administration or withdrawal at the indicated time points. (F) Representative immunofluorescent images (left panel) and quantification (right panel) of CDCP1+tdTomato+ cells 24 h post-tamoxifen injection in FCT mice (n = 3). Arrowheads indicate the CDCP1+tdTomato+ cells. (G) Representative images (left panels) and quantification (right panel) of tdTomato labeling in FCT primary tumors (n = 3) versus relapsed tumors after FOLFOX withdrawal (n = 3). Values shown are mean ± SD. P-values were calculated by two-tailed t test unless otherwise indicated. **p < 0.05. Bar: 50 μm. Underlying data are available in S1 Data. Apc, adenomatous polyposis coli; CDCP1, CUB-domain–containing protein 1; CRC, colorectal carcinoma; CSC, cancer stem cell; Dox, doxycycline; Fapbl, liver-type fatty-acid–binding protein; FCT, Fabpl4X@132-rtTA-3×(IRES-Apc.3374):tet-KrasG12D:CDCP1-creERT2:Rosa26 CAG-loxP-stop-loxP-tdTomato; FOLFOX, folinic acid + fluorouracil + oxaliplatin; HE, hematoxylin–eosin; IRES, internal ribosome entry site; Kras, Kirsten rat sarcoma viral oncogene homolog; muKras, mutant Kras; qPCR, quantitative PCR; Raf, murine leukemia viral oncogene homolog; Ras, rat sarcoma viral oncogene homolog; RBD, Ras-binding d [file pbio.3000425.s002.tif]

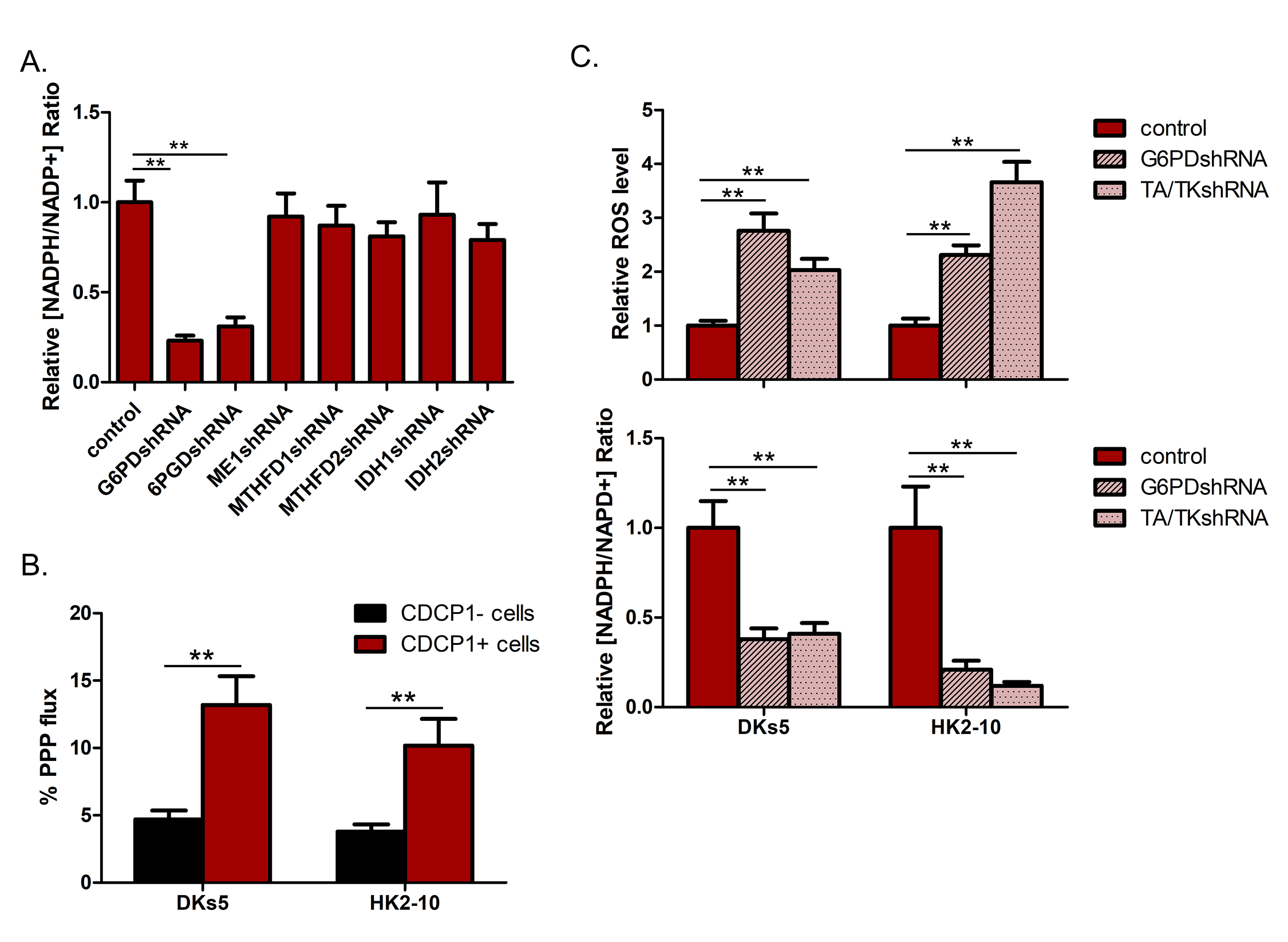

Supplement: S3 Fig — (A) The ratios of [NADPH/NAPD+] in CRC108-derived CDCP1+ cells transfected with control shRNA or shRNA targeting G6PD, 6PGD, ME1, MTHFD1, MTHFD2, IDH1, or IDH2, respectively. (B) Percentage of central carbon flux from glucose to lactate flowing through the PPP in CDCP1+ and CDCP1– fractions isolated from DKs5 and HK2-10 cells. Flux was determined from the relative enrichment of doubly versus singly [13C]-labeled lactate, pyruvate, and 3-phosphoglycerate, as measured using negative mode LC-MS of extracts from cells fed with [1,2-13C]-glucose. (C) ROS levels and the ratios of [NADPH/NAPD+] in DKs5- or HK2-10–derived CDCP1+ cells with G6PD or TK/TA KD. All experiments were independently repeated three times in triplicate. Values shown are mean ± SD. A two-tailed unpaired t test was used to compare experimental groups. **p < 0.05. Underlying data are available in S1 Data. CDCP1, CUB-domain–containing protein 1; CRC, colorectal carcinoma; CSC, cancer stem cell; G6PD, glucose-6-phosphate dehydrogenase; IDH, isocitrate dehydrogenase; KD, knockdown; Kras, Kirsten rat sarcoma viral oncogene homolog; LC-MS, liquid chromatography-triple quadrupole mass spectrometry; ME1, malic enzyme 1; MTHFD, methylenetetrahydrofolate dehydrogenase; muKras, mutant Kras; PPP, pentose phosphate pathway; ROS, reactive oxygen species; shRNA, short hairpin RNA; TA, transaldolase; TK, transketolase; 6PGD, 6-phosphogluconate dehydrogenase. (TIF) [file pbio.3000425.s003.tif]

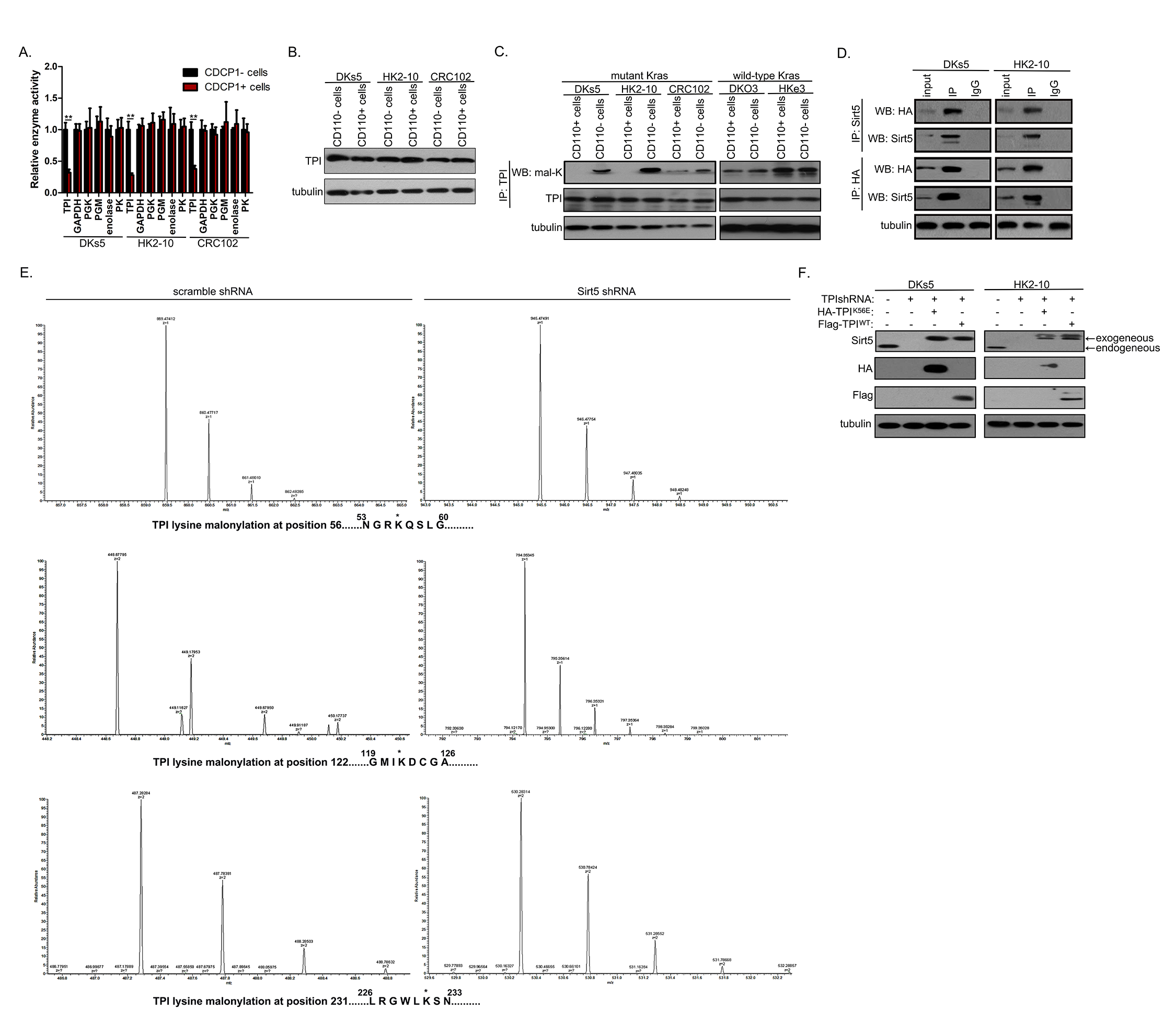

Supplement: S4 Fig — (A) The enzyme activities of TPI, GAPDH, PGK, PGM, enolase, and PK were compared between CDCP1– and CDCP1+ fractions from DKs5, HK2-10, and CRC102 cells. The experiments were independently repeated three times in triplicate. (B) The expression level of endogenous TPI was compared between CDCP1– and CDCP1+ fractions from DKs5, HK2-10, and CRC102 cells. (C) TPI malonylation was compared between CDCP1– and CDCP1+ fractions from wtKras and muKras CRC cells. (D) The HA-H158Y mutant was transfected into DKs5- or HK2-10–derived CDCP1+ cells. The association of endogenous TPI with HA-H158Y was determined by co-IP. (E) Annotation of a representative tandem mass spectrum of trypsin-digested TPI showing malonylation of K56, K122, and K231 upon Sirt5 KD in CRC108-derived CDCP1+ cells. (F) The indicated HA or Flag-tagged TPI proteins were overexpressed in DKs5-derived CDCP1+ cells with stable TPI KD. The presence of ectopically expressed and endogenous proteins was verified by western blot. Values shown are mean ± SD. A two-tailed unpaired t test was used to compare experimental groups. Underlying data are available in S1 Data. CDCP1, CUB-domain–containing protein 1; CRC, colorectal carcinoma; GAPDH, glyceraldehyde -3-phosphate dehydrogenase; HA, hemagglutinin; IP, immunoprecipitation; KD, knockdown; Kras, Kirsten rat sarcoma viral oncogene homolog; muKras, mutant Kras; PGK, phosphoglycerate kinase; PGM, phosphoglucomutase; PK, pyruvate kinase; Sirt5, silent mating type information regulation 2 homolog 5; TPI, triosephosphate isomerase; wtKras, wild-type Kras. (TIF) [file pbio.3000425.s004.tif]

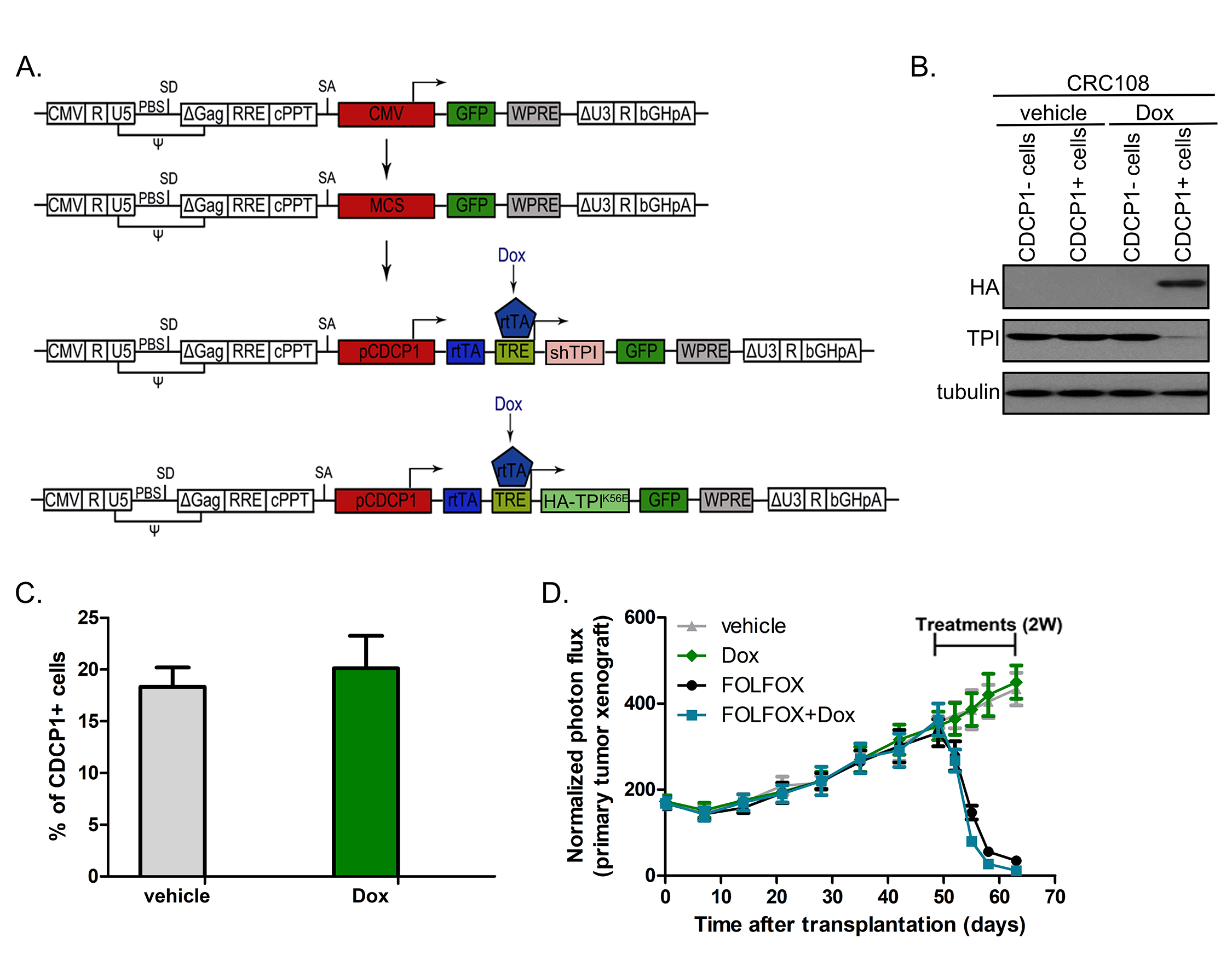

Supplement: S5 Fig — (A) Schematic representation of the parental (upper), intermediate (middle), and final dual-promoter (lower) lentiviral vectors. (B–C) CRC108 cells labeled with GFP were transfected with the dual-promoter lentiviral vectors in S5A Fig. HA-TPI and TPI expression in CDCP1– and CDCP1+ subpopulations were analyzed at 24 h after Dox treatment (1 μg/ml) (B); the frequency of CDCP1+ cells was analyzed at 72 h after Dox treatment (1 μg/ml) (C). The experiments were independently repeated three times in triplicate. (D) Upon visible tumor formation after orthotopic implantation into 4-week–old female NOG mice, animals were randomized to receive FOLFOX (twice weekly for 2 weeks) in the presence (n = 35) or absence (n = 33) of Dox. Tumor growth and regression were monitored after implantation (n = 3 per time point). Values shown are mean ± SD. A two-tailed unpaired t test was used to compare experimental groups. **p < 0.05. Underlying data are available in S1 Data. CDCP1, CUB-domain–containing protein 1; CRC, colorectal carcinoma; CSC, cancer stem cell; Dox, doxycycline; FOLFOX, folinic acid + fluorouracil + oxaliplatin; GFP, green fluorescent protein; HA, hemagglutinin; Kras, Kirsten rat sarcoma viral oncogene homolog; muKras, mutant Kras; NOG, nonobese diabetic (NOD)/Shi-scid Il2rgnull; PPP, pentose phosphate pathway; TPI, triosephosphate isomerase. (TIF) [file pbio.3000425.s005.tif]

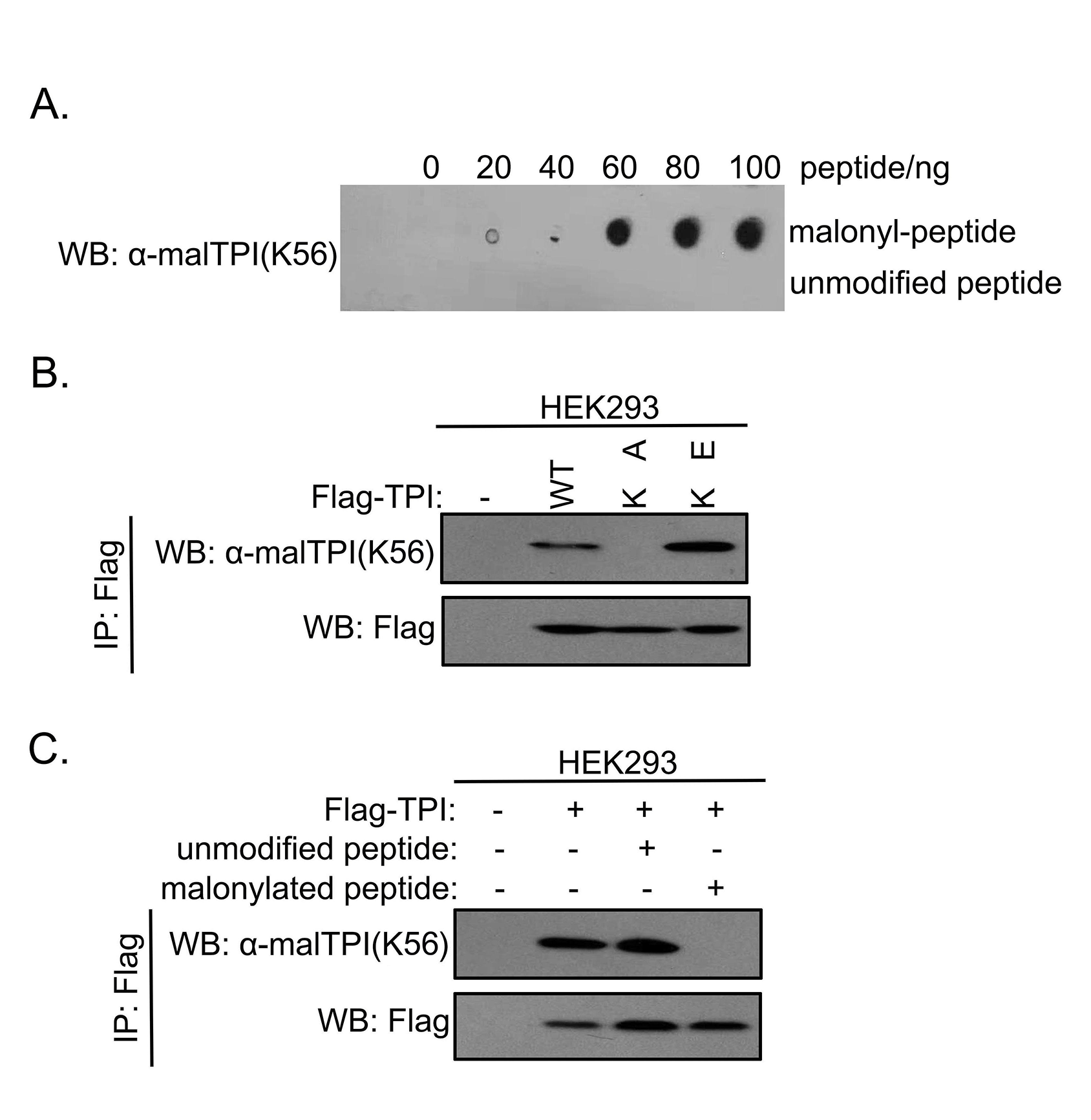

Supplement: S6 Fig — (A) Specificity of antibody against malonylated K56 of TPI was determined by dot blot assay. Nitrocellulose membrane was spotted with different amounts of malonyl-K56 peptide or unmodified peptide and detected with a site-specific antibody against K56 malonylation [α-malTPI(K56)]. (B) Immunoprecipitated Flag-tagged WT TPI or its K56A/K56E mutants were detected by the α-malTPI(K56) antibody. (C) Malonylated K56 peptide, but not the unmodified peptide, competed with malonylated TPI. malTPI, malonylated TPI; TPI, triosephosphate isomerase; WT, wild type. (TIF) [file pbio.3000425.s006.tif]
